# Supplementary figures and images for: The Genetics of Bene Israel from India Reveals Both Substantial Jewish and Indian Ancestry
Source: PLoS One. 2016 Mar 24;11(3):e0152056. doi: 10.1371/journal.pone.0152056 (PMC4806850; doi:10.1371/journal.pone.0152056)

**A**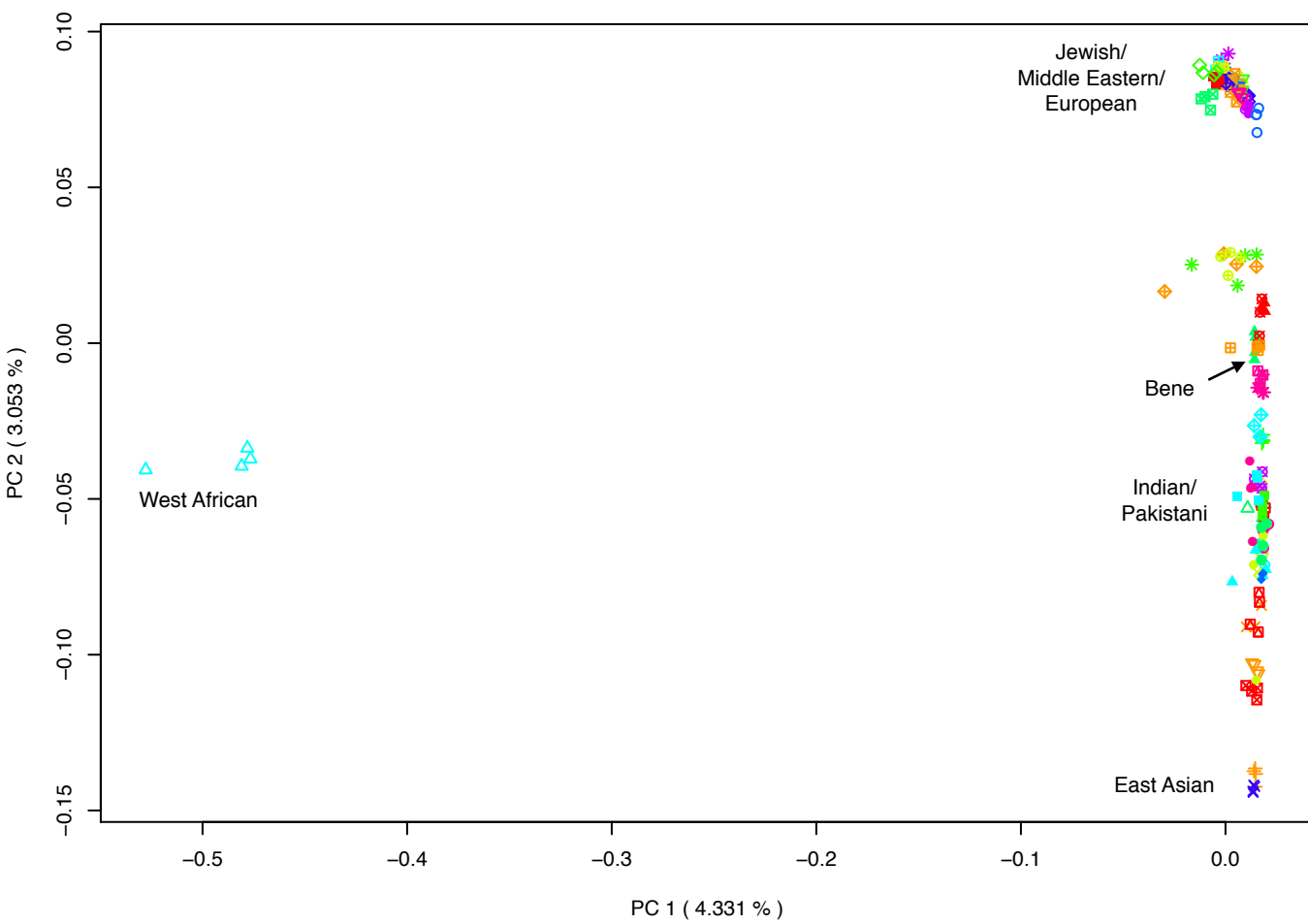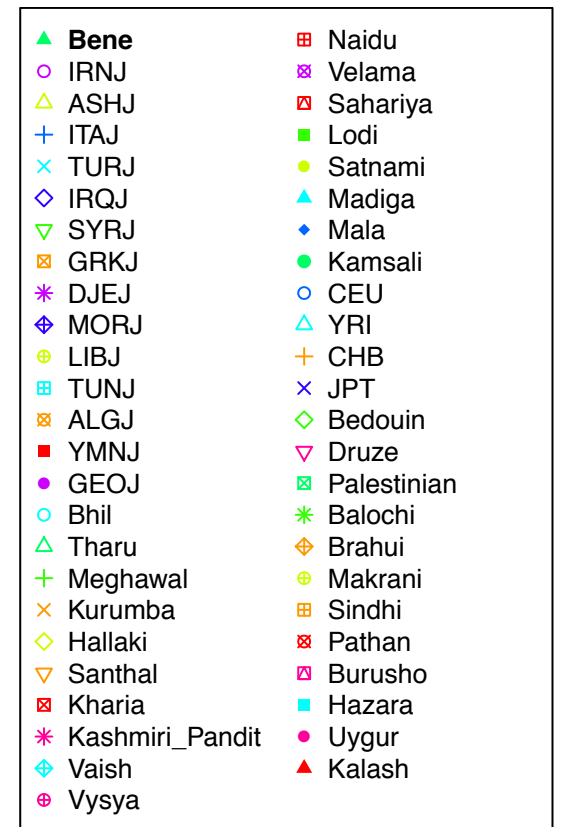**B**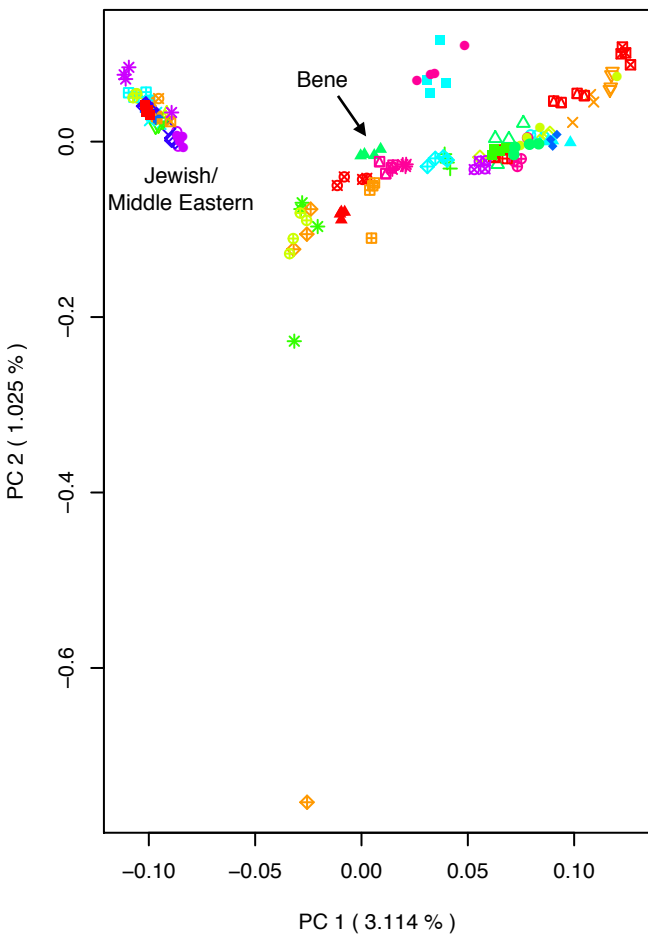**C**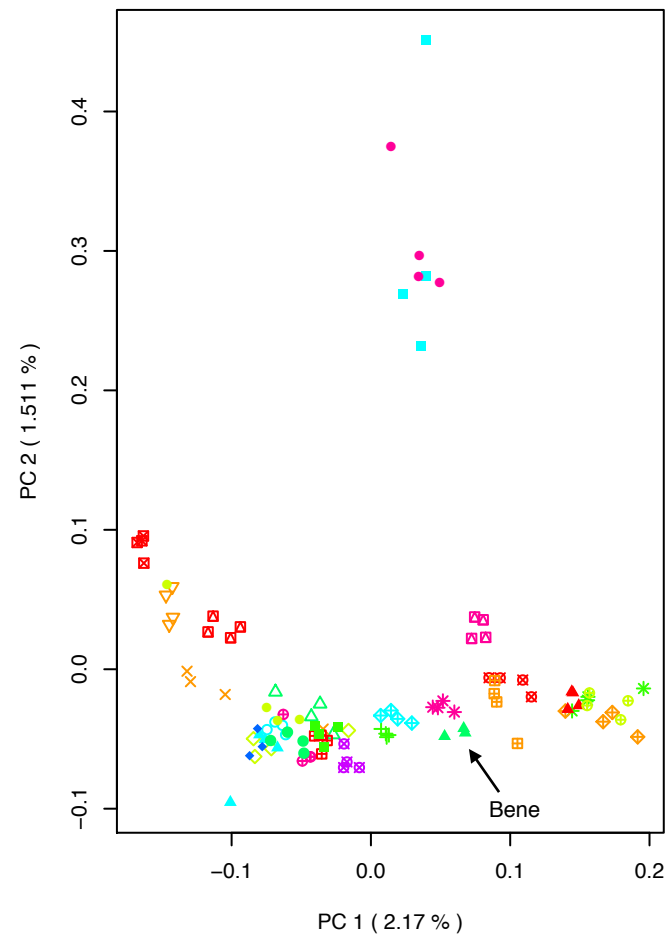**D**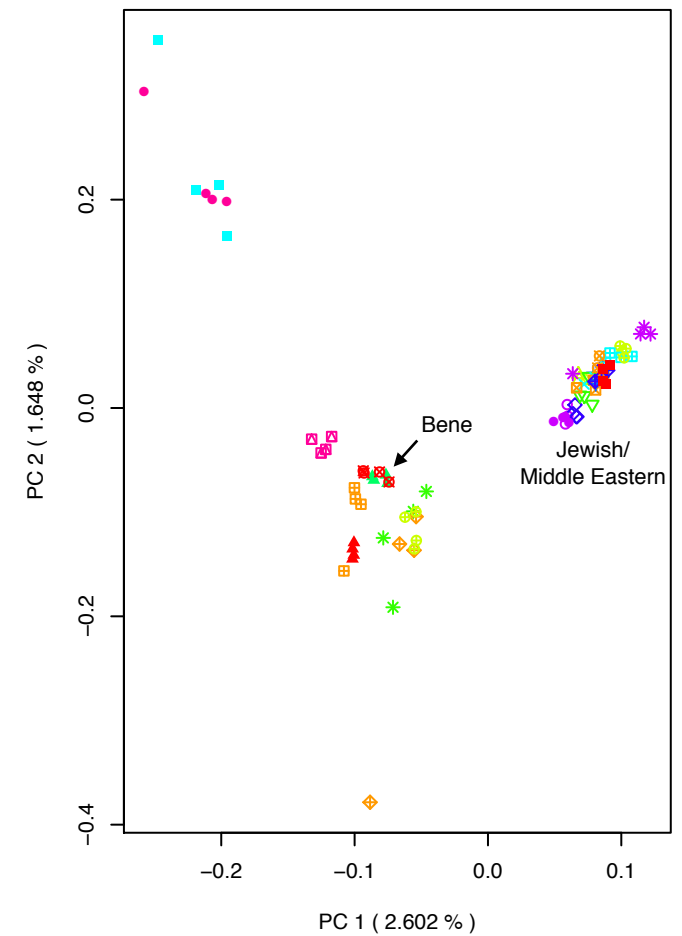

Supplement: S1 Fig — In this figure, and in order to avoid bias due to the larger number of Jewish samples, we repeated the analysis as in the main text (Fig 1) while limiting the number of samples from each population to be no more than four. (PDF) [file pone.0152056.s001.pdf]

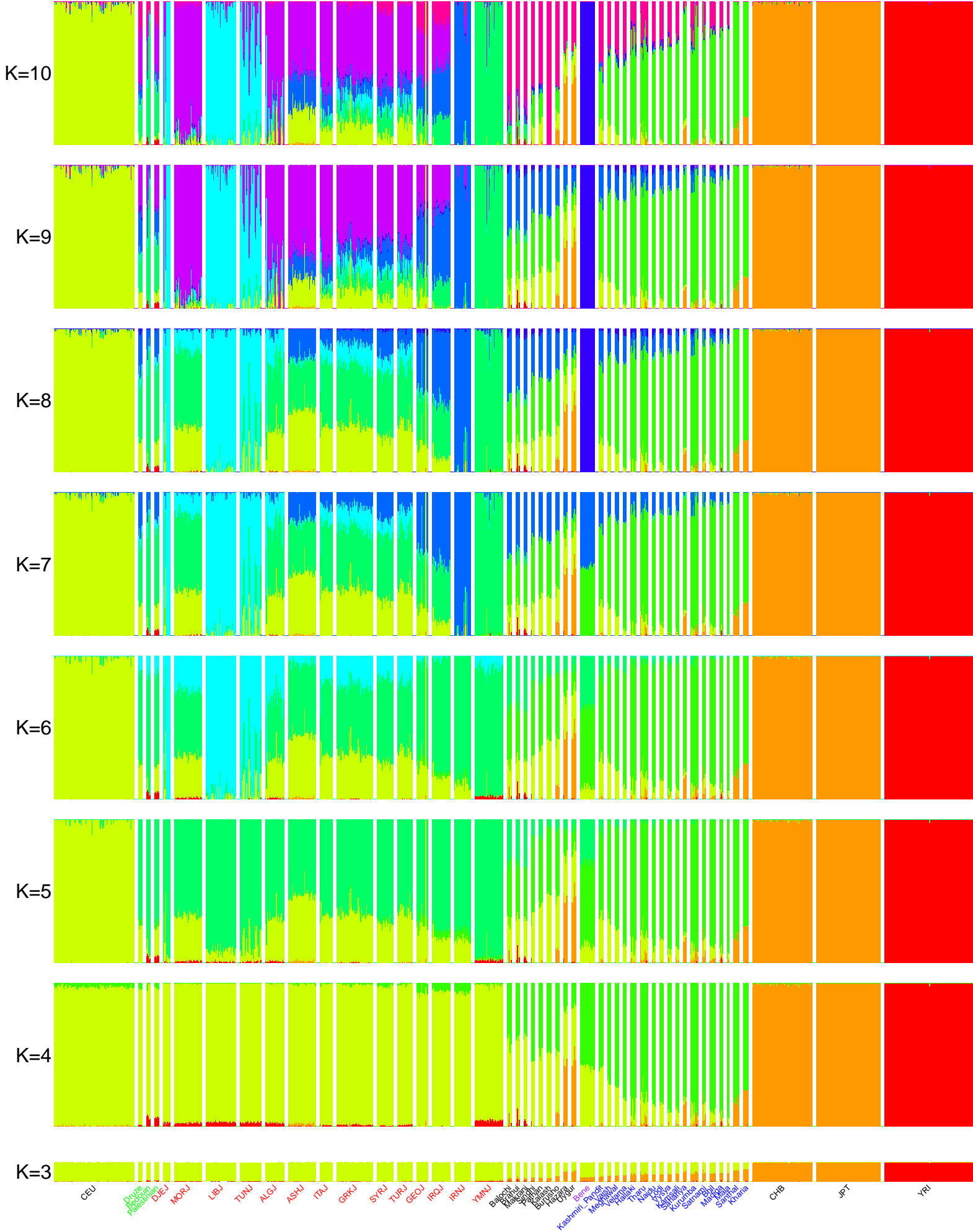

Supplement: S3 Fig — We colored some of the populations groups: Bene Israel (purple), Jews (red), Indians (blue) and Middle-Eastern (green). (PDF) [file pone.0152056.s003.pdf]

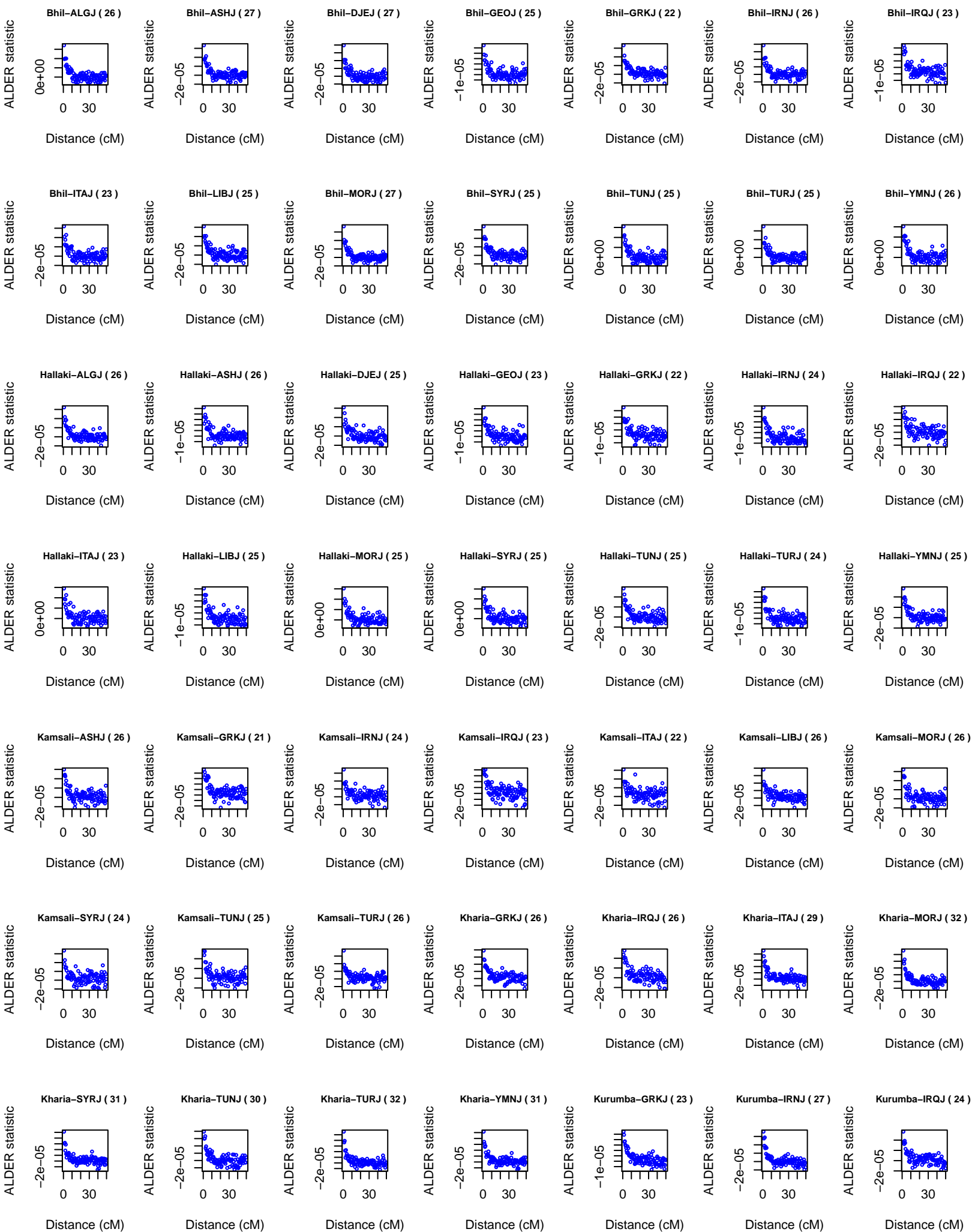

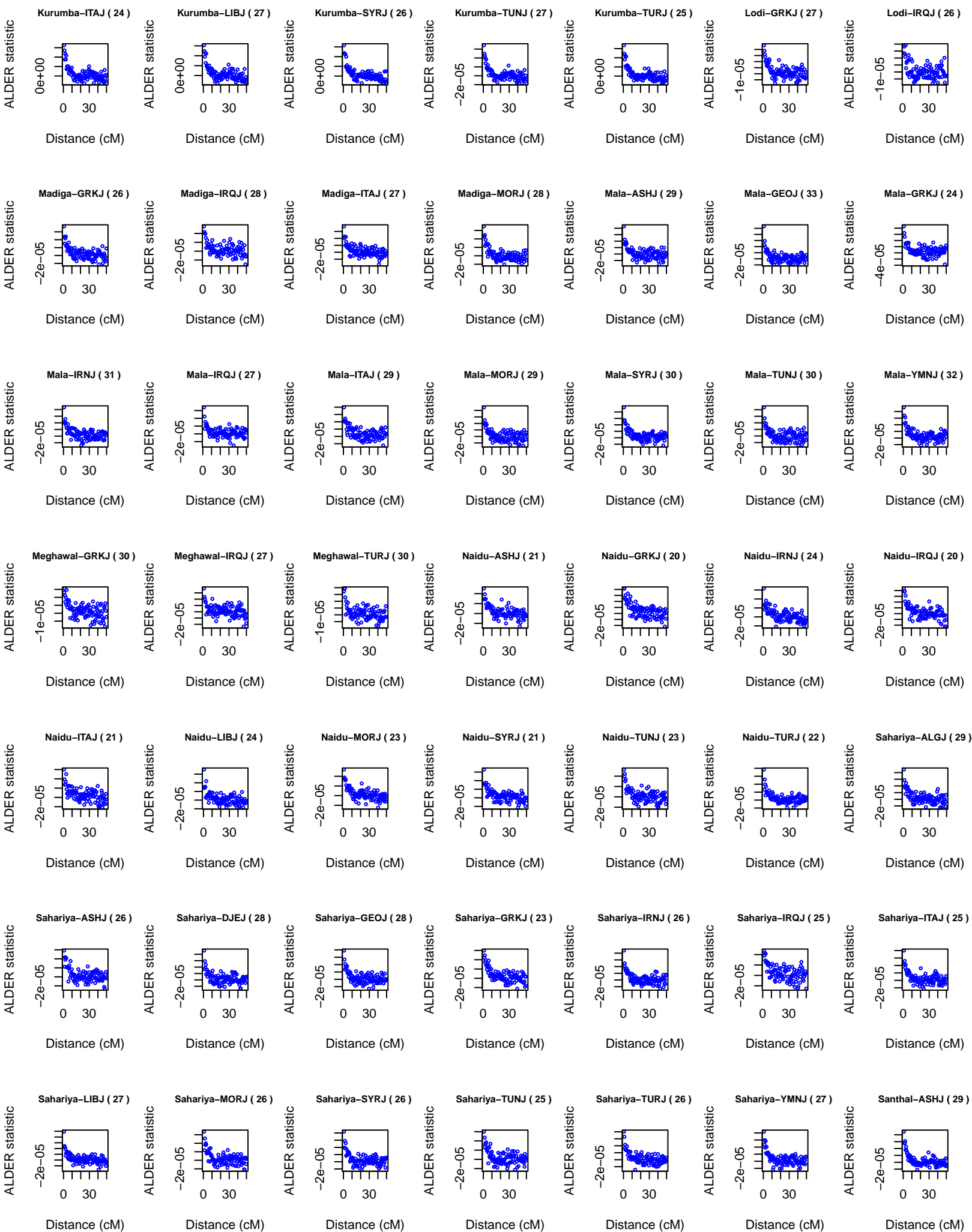

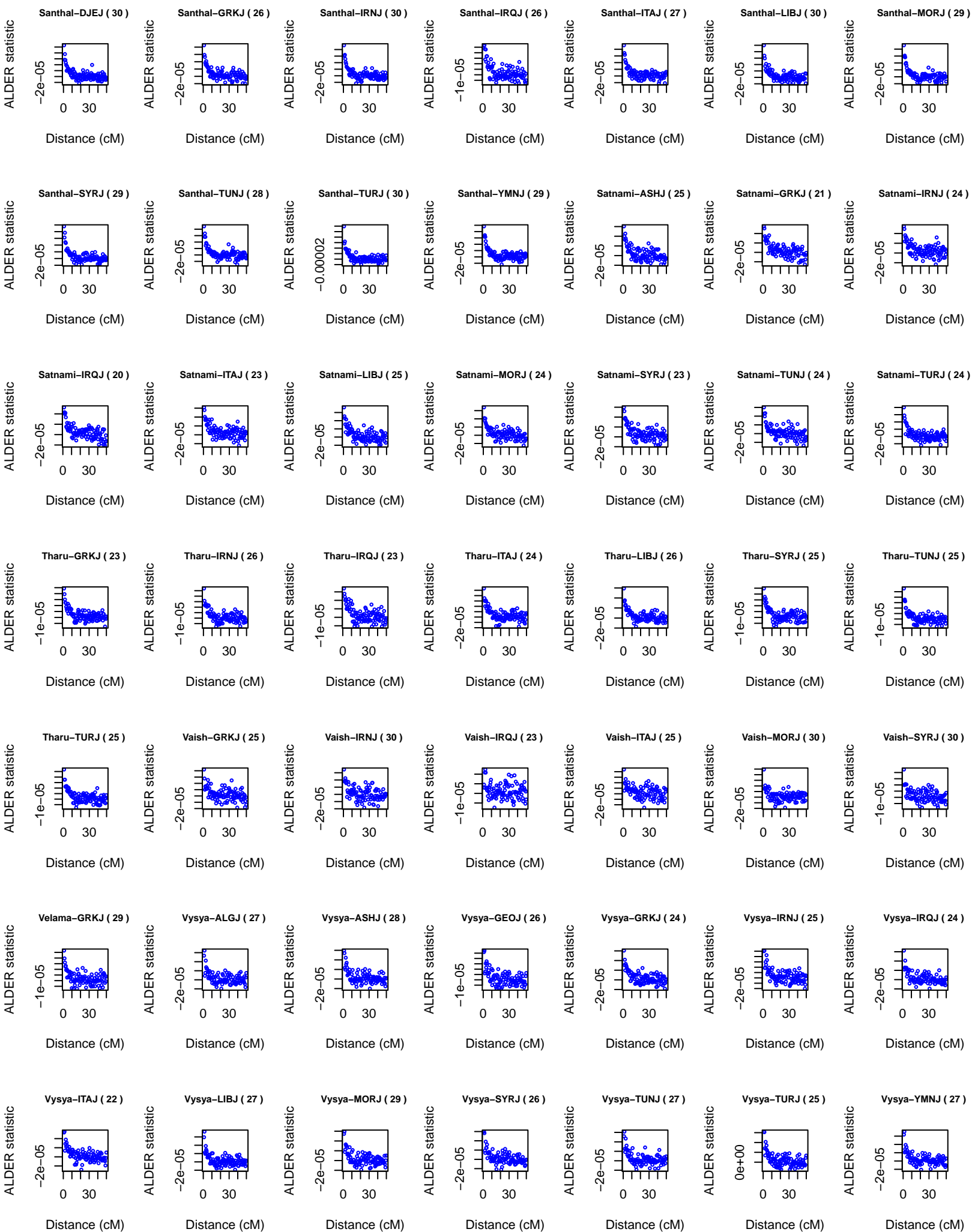

Supplement: S6 Fig — The number above each plot represent the predicted time (in generations) since admixture. See S3 Table for more details. (PDF) [file pone.0152056.s006.pdf]

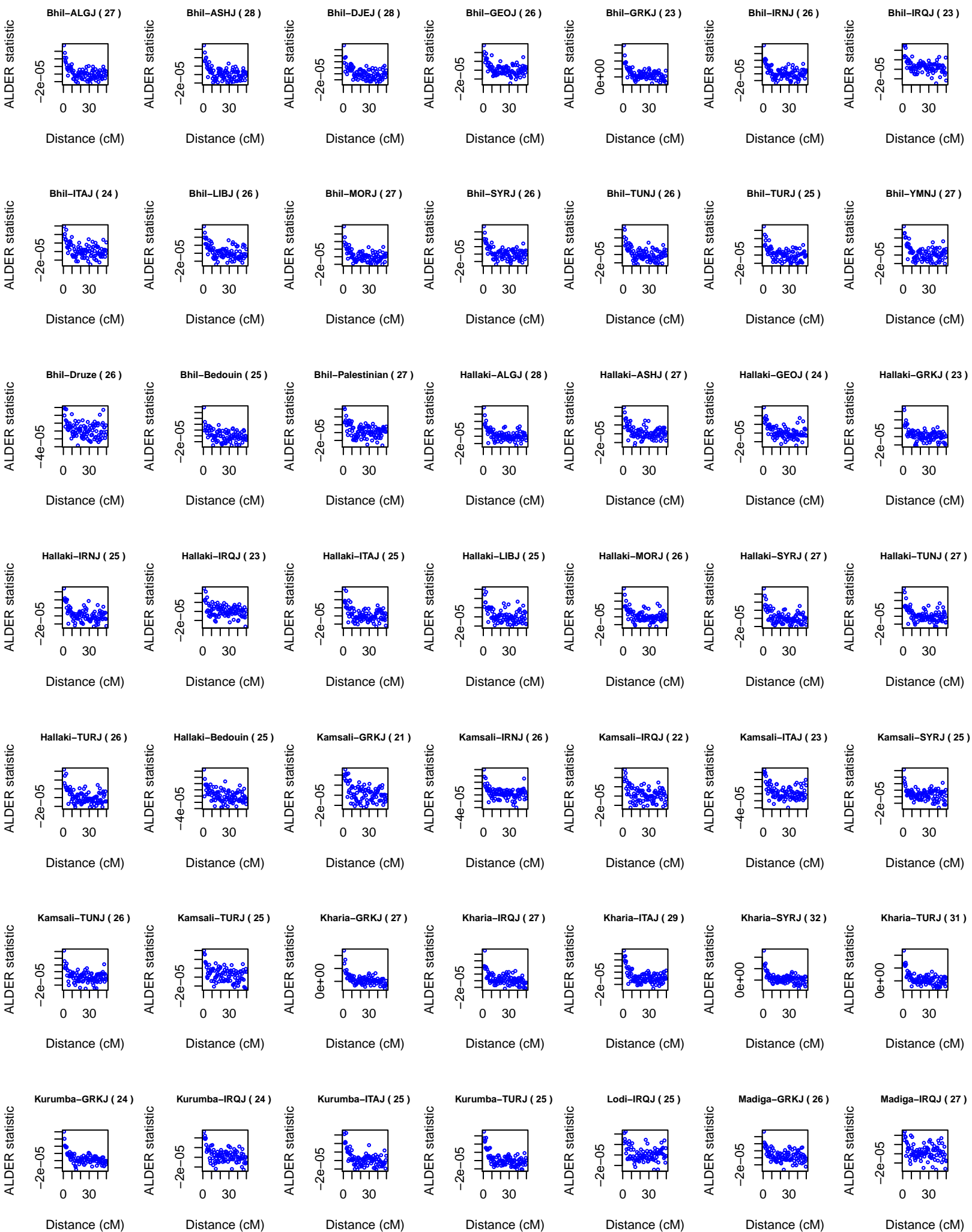

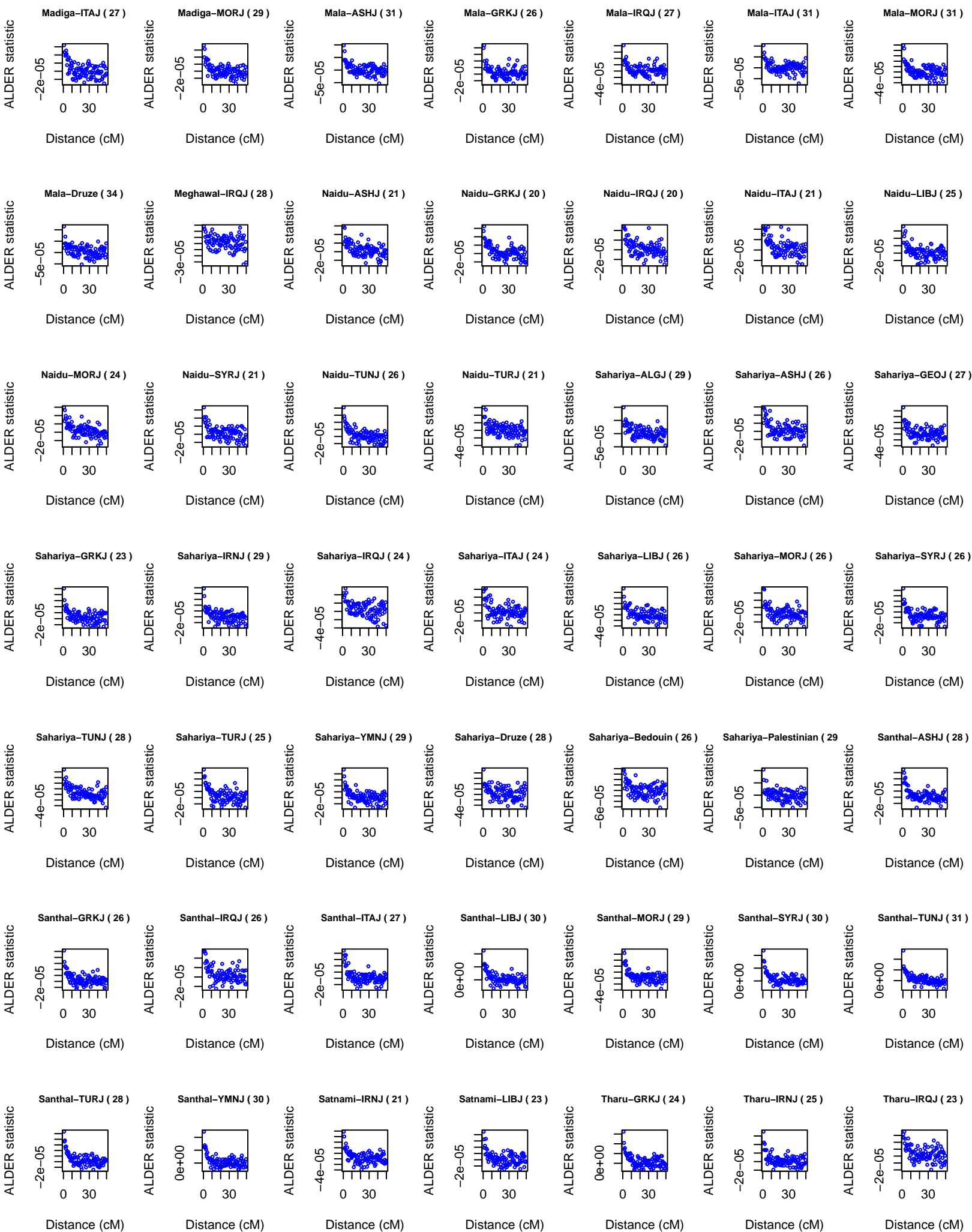

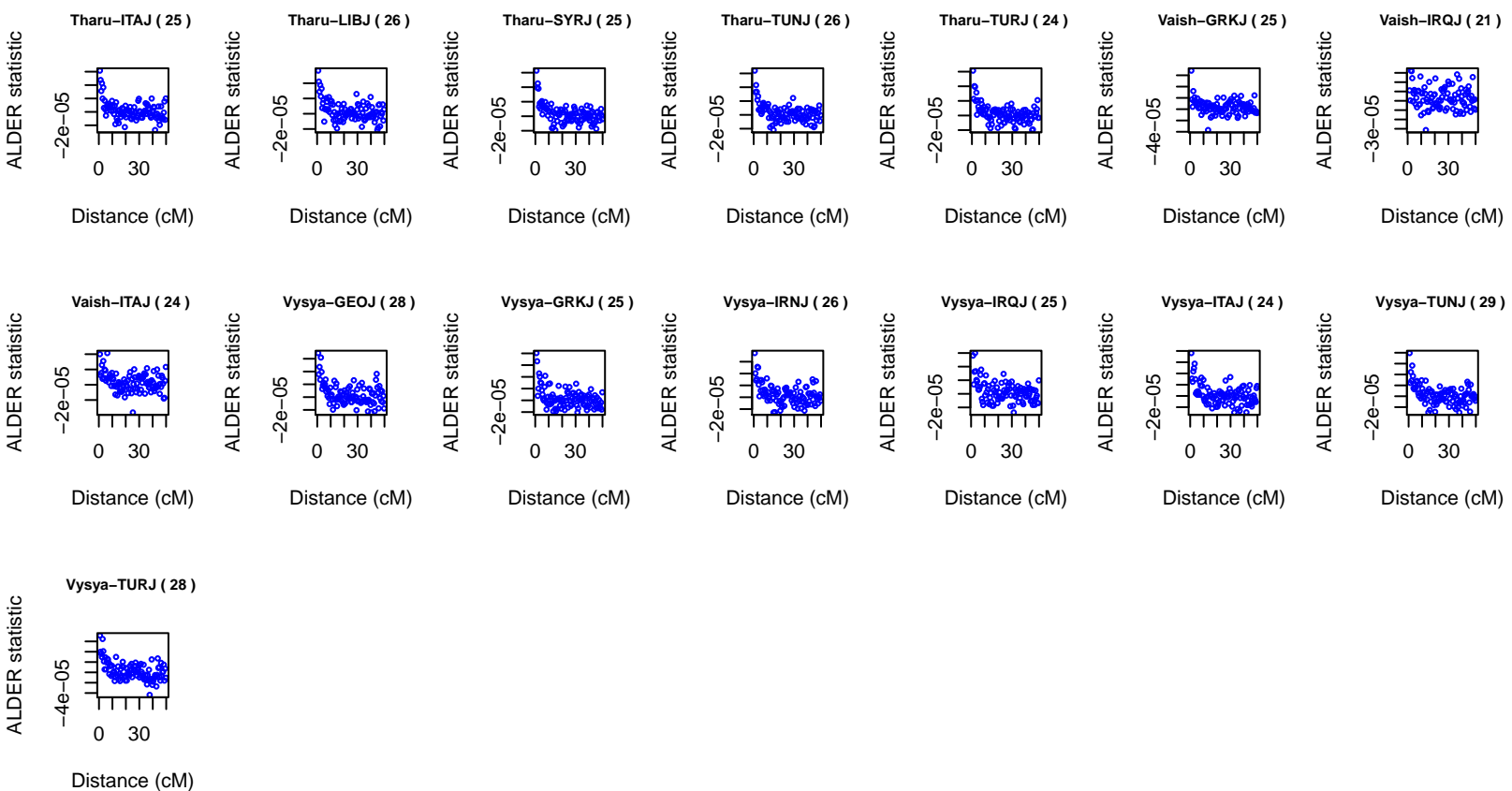

Supplement: S7 Fig — The number above each plot represent the predicted time (in generations) since admixture. See S4 Table for more details. (PDF) [file pone.0152056.s007.pdf]

# F4 Test

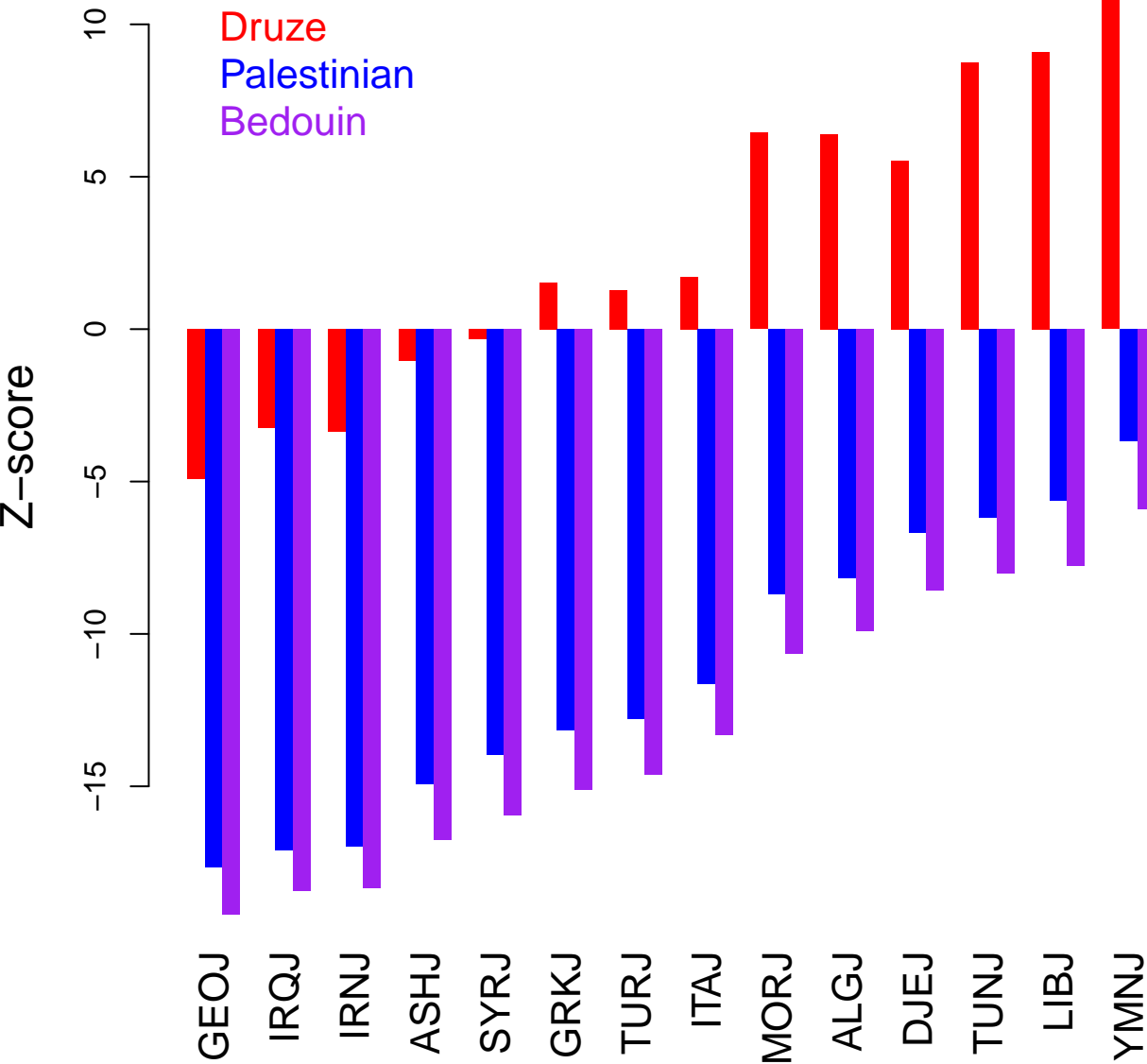

Supplement: S8 Fig — We performed f4(YRI, Bene Israel; J, ME) tests where J and ME are Jewish and Middle-Eastern population, respectively. Negative Z-scores suggest that ME is an outgroup populations for (Bene Israel, J). (PDF) [file pone.0152056.s008.pdf]

Het

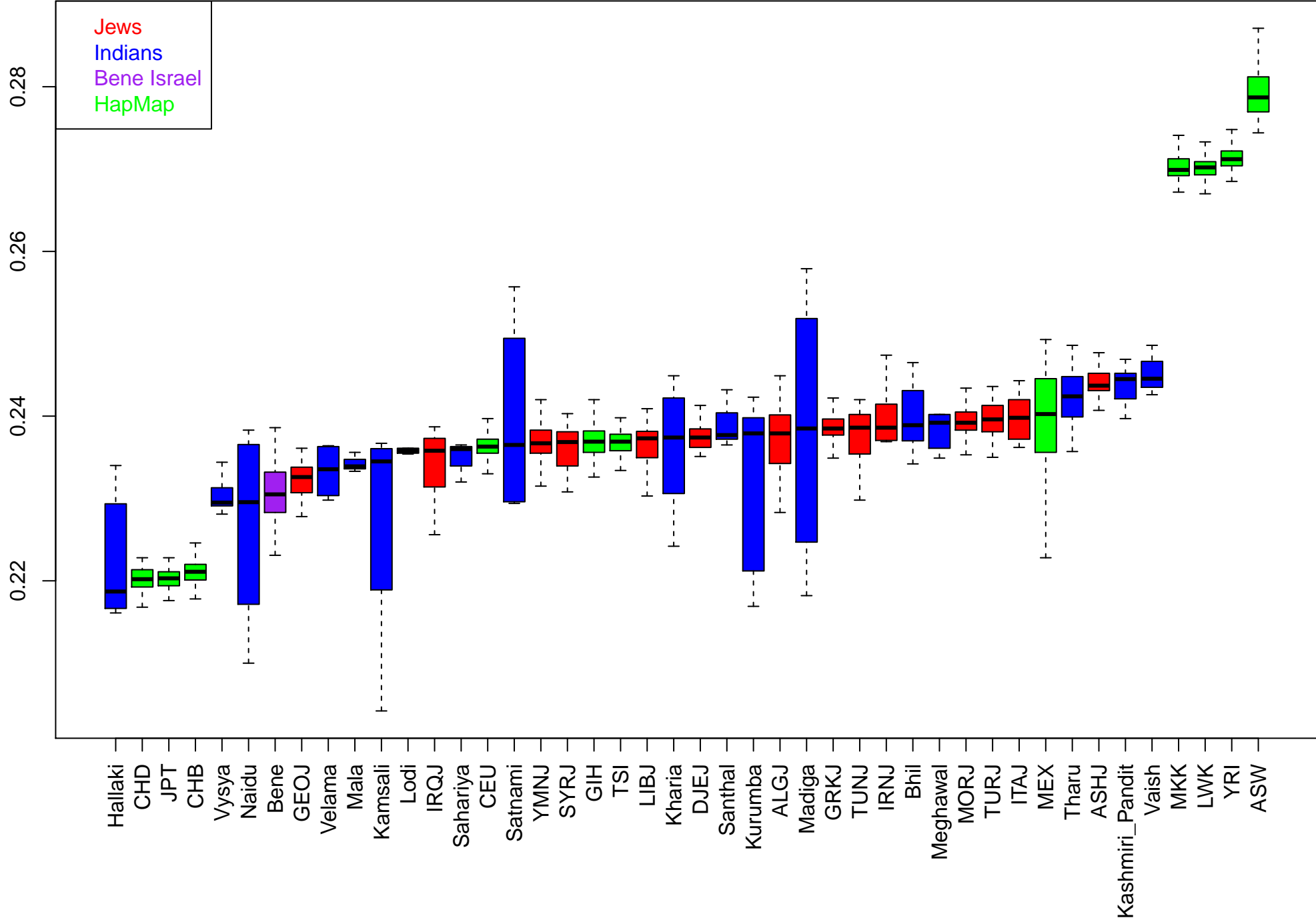

Supplement: S9 Fig — The larger variance in Het values in some Indian populations is due to smaller sample size. (PDF) [file pone.0152056.s009.pdf]

K=3

ASHU

INDJ

Bene

Sheba

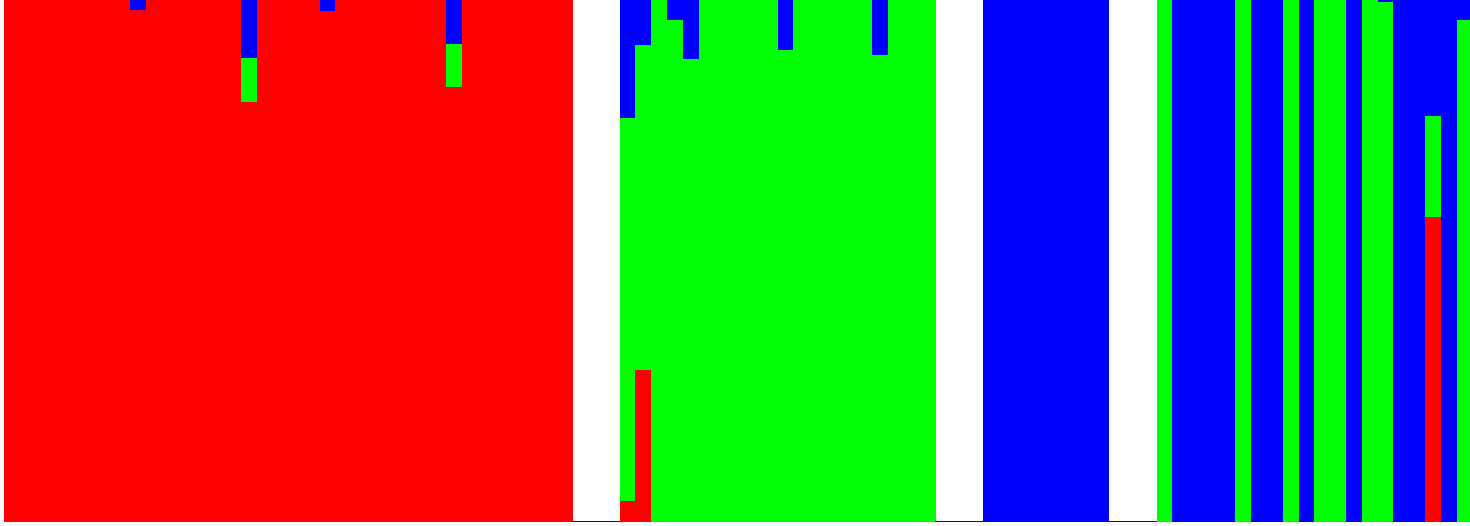

Supplement: S10 Fig — This was carried out to divide the Sheba samples into Bene Israel and Cochin Jews (Materials and Methods). (PDF) [file pone.0152056.s010.pdf]

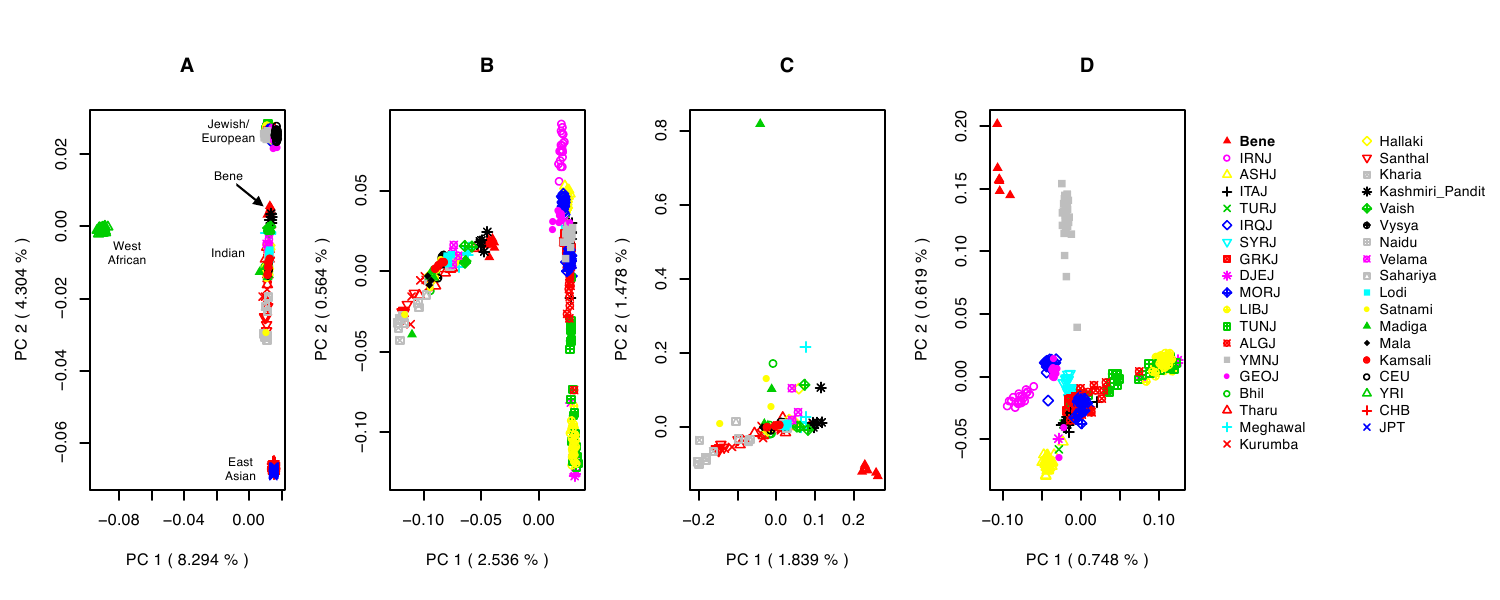

Supplement: S11 Fig — This was carried out to assign the Sheba samples into Bene Israel and Cochin Jews (Materials and Methods). (PNG) [file pone.0152056.s011.png]

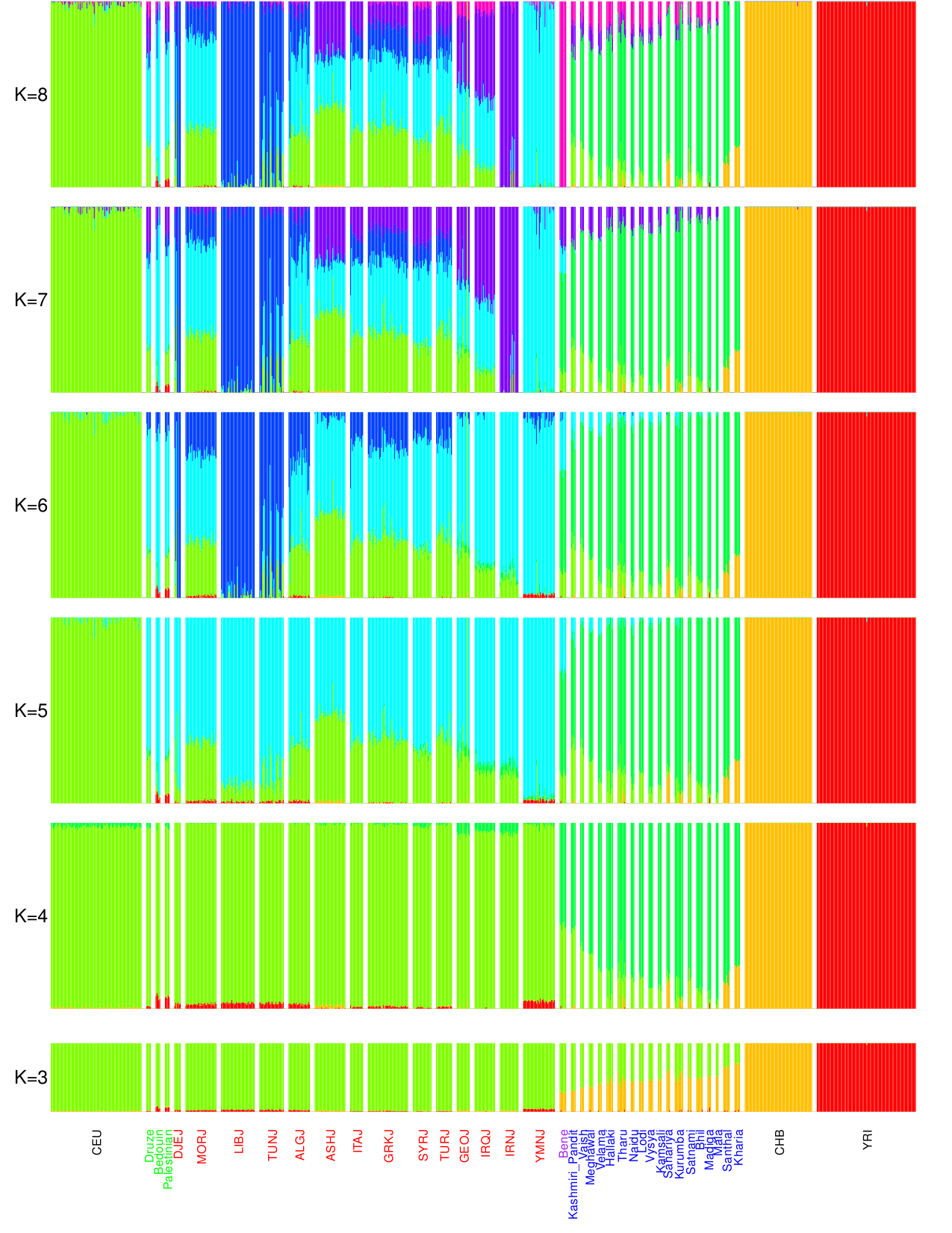

Supplement: S12 Fig — We repeated the analysis similar to that in the main text (Fig 1) with Bene Israel samples being only those collected in Ramla and not in Sheba Medical Center (see Materials and Methods). (PNG) [file pone.0152056.s012.png]

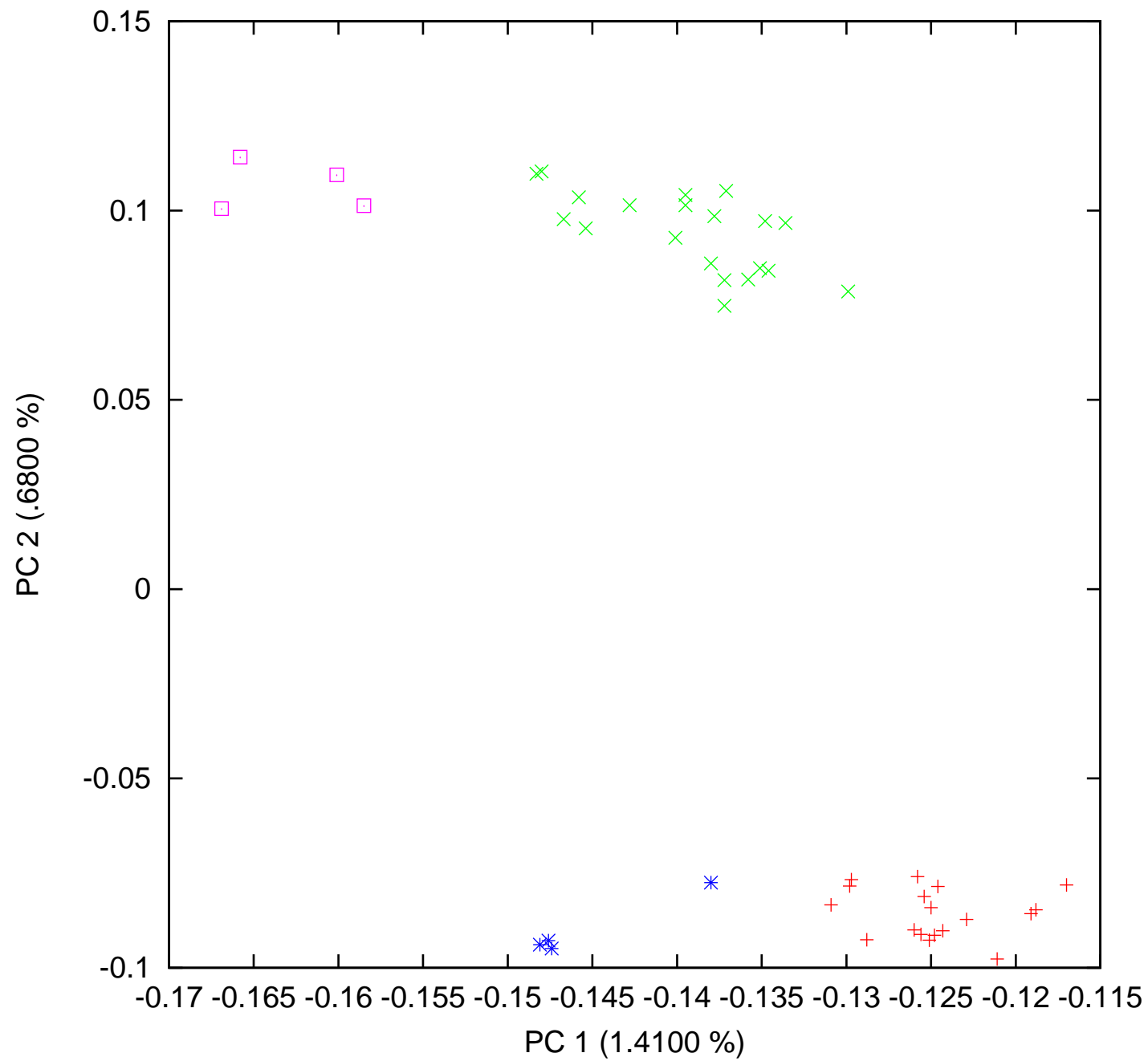

Supplement: S13 Fig — We repeated the analysis similar to that in the main text (Fig 2) with Bene Israel samples being only those collected in Ramla and not in Sheba Medical Center (see Materials and Methods). (PDF) [file pone.0152056.s013.pdf]

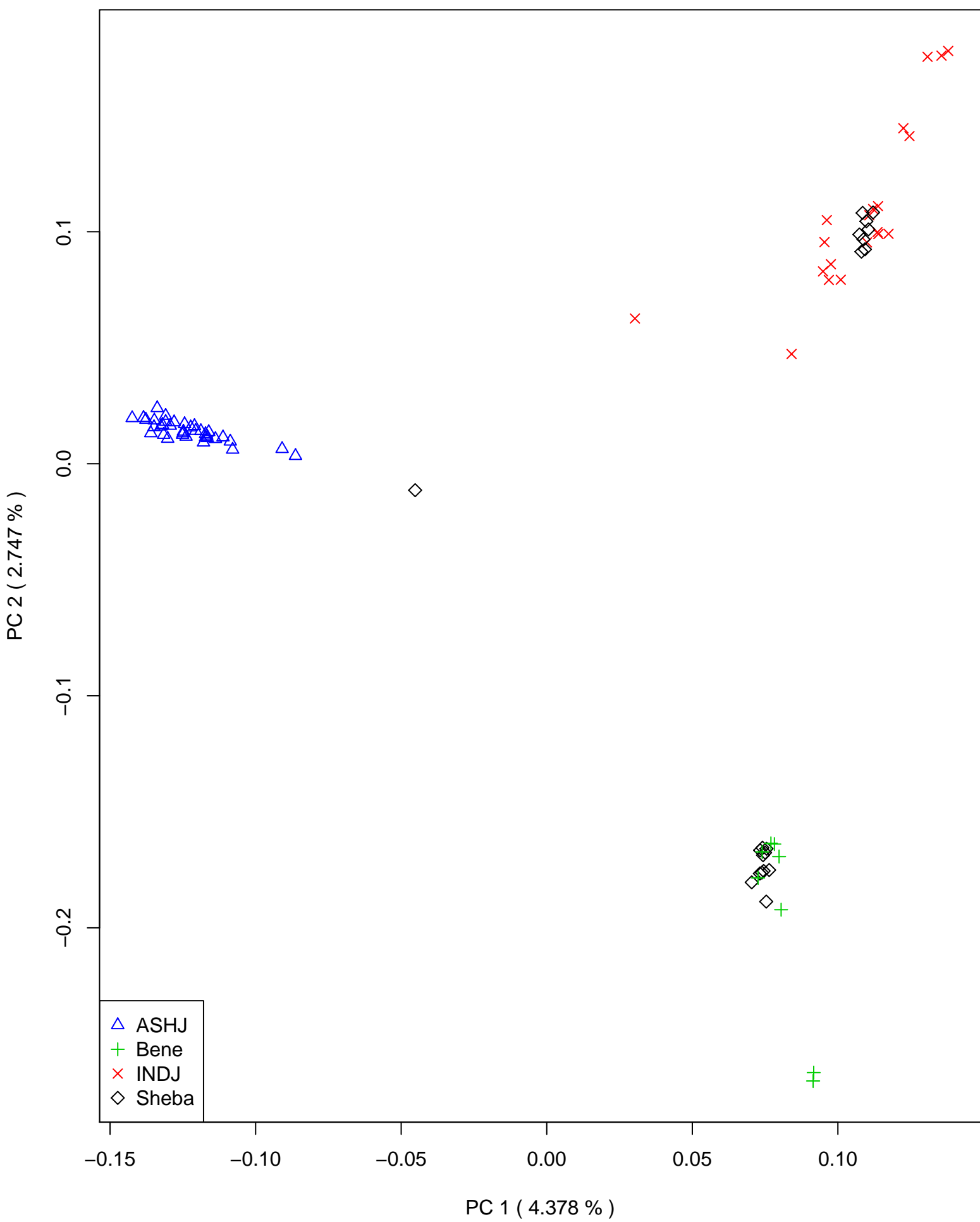

Supplement: S14 Fig — (PDF) [file pone.0152056.s014.pdf]
