# Supplementary material for: The Genetics of Bene Israel from India Reveals Both Substantial Jewish and Indian Ancestry
Source: PLoS One. 2016 Mar 24;11(3):e0152056. doi: 10.1371/journal.pone.0152056 (PMC4806850; doi:10.1371/journal.pone.0152056)
Supplement: S2 Fig — (PDF) [file pone.0152056.s002.pdf]

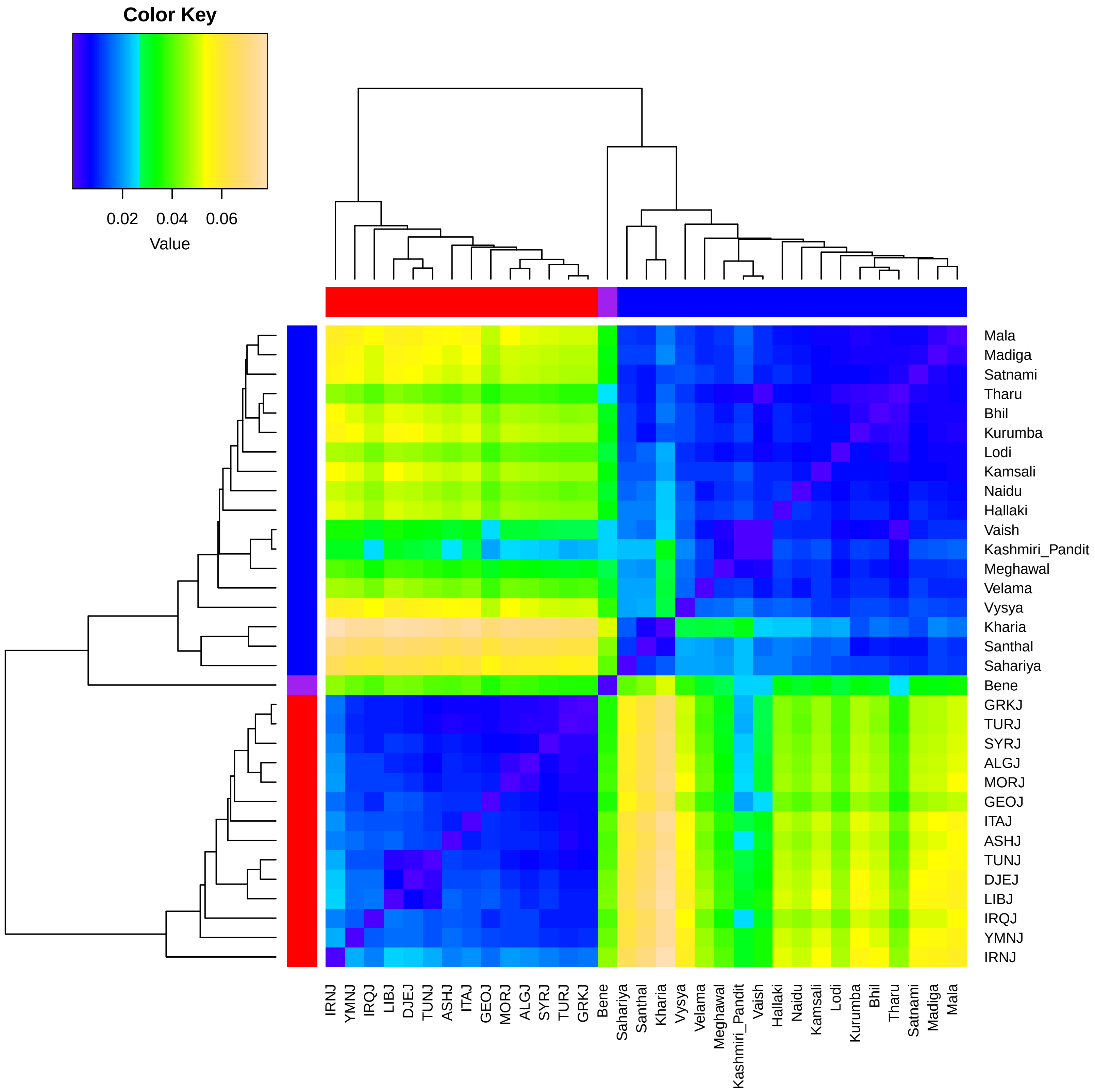

Color Key
0.02
0.04
0.06
Value
Mala
Madiga
Satnami
Tharu
Bhil
Kurumba
Lodi
Kamsali
Naidu
Hallaki
Vaish
Kashmiri_Pandit
Meghawal
Velama
Vysya
Kharia
Santhal
Sahariya
Bene
GRKJ
TURJ
SYRJ
ALGJ
MORJ
GEOJ
ITAJ
ASHJ
TUNJ
DJEJ
LIBJ
IRQJ
YMNJ
IRNJ
