# Supplementary material for: The Genetics of Bene Israel from India Reveals Both Substantial Jewish and Indian Ancestry
Source: PLoS One. 2016 Mar 24;11(3):e0152056. doi: 10.1371/journal.pone.0152056 (PMC4806850; doi:10.1371/journal.pone.0152056)
Supplement: S4 Fig — Heat map of IBD sharing between Indian and Jewish populations for (A) Jewish and Indians populations and (B) for Jewish, Indian, Pakistani and Middle-Eastern populations. Entry (i,j) in the heat map presents the mean IBD sharing between individuals from populations i and j. Values on the diagonal represent IBD within populations. (PDF) [file pone.0152056.s004.pdf]

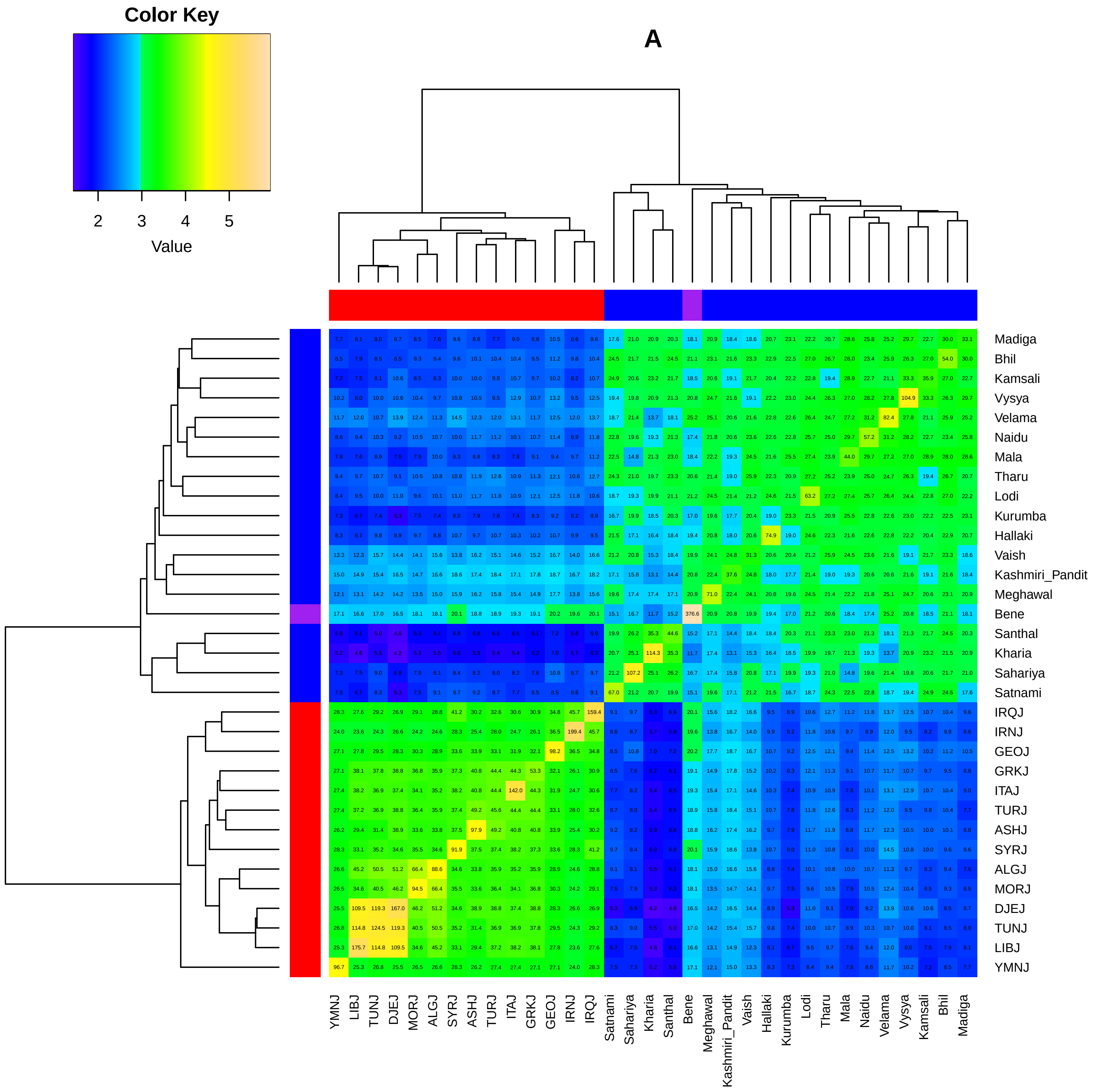

Color Key
2
3
4
5
Value
A
Madiga
Bhil
Kamsali
Vysya
Velama
Naidu
Mala
Tharu
Lodi
Kurumba
Hallaki
Vaish
Kashmiri_Pandit
Meghawal
Bene
Santhal
Kharia
Sahariya
Satnami
IRQJ
IRNJ
GEOJ
GRKJ
ITAJ
TURJ
ASHJ
SYRJ
ALGJ
MORJ
DJEJ
TUNJ
LIBJ
YMNJ
YMNJ
LIBJ
TUNJ
DJEJ
MORJ
ALGJ
SYRJ
ASHJ
TURJ
ITAJ
GRKJ
GEOJ
IRNJ
IRQJ
Satnami
Sahariya
Kharia
Santhal
Bene
Meghawal
Kashmiri_Pandit
Vaish
Hallaki
Kurumba
Lodi
Tharu
Mala
Naidu
Velama
Vysya
Kamsali
Bhil
Madiga

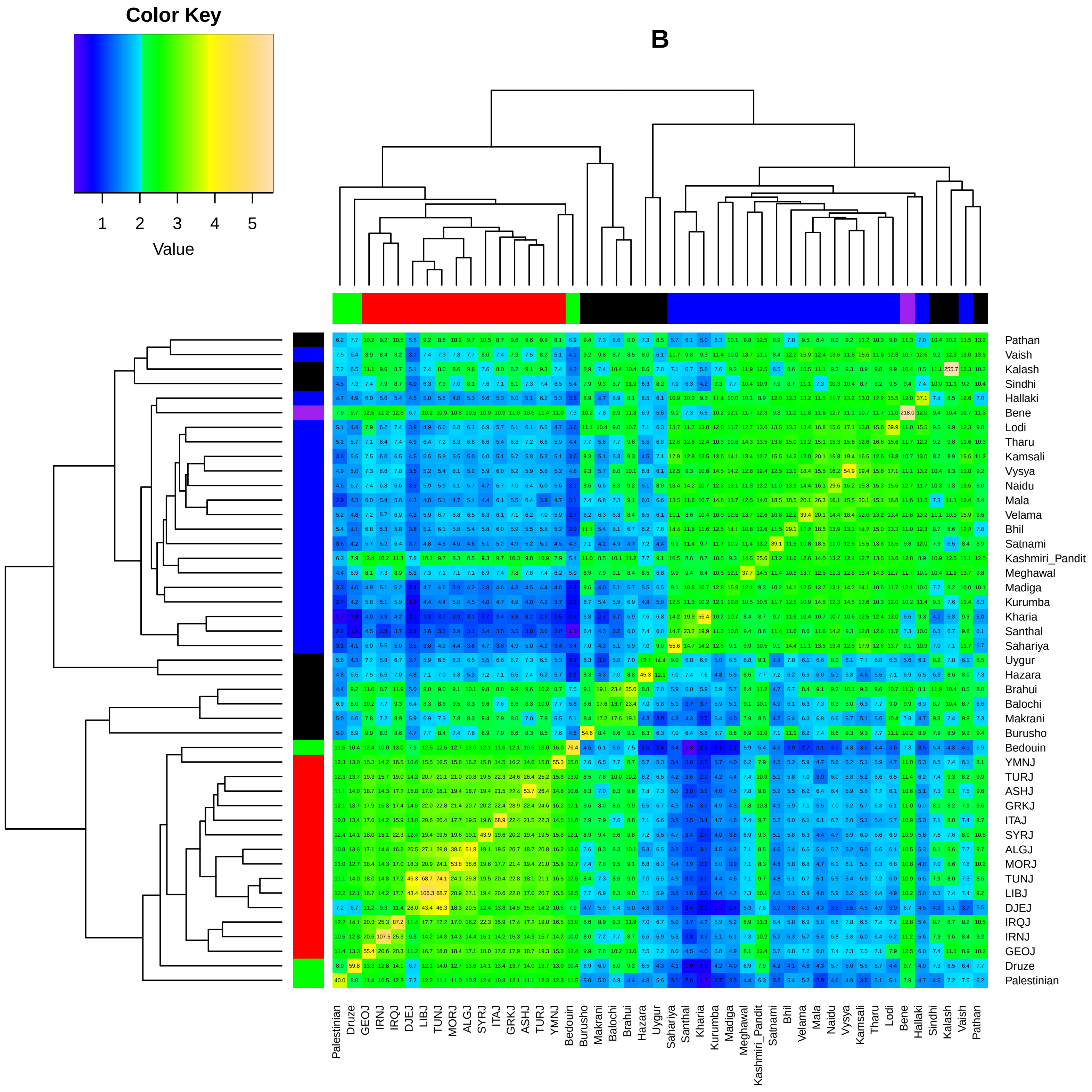
Color Key
1
2
3
4
5
Value
B
Pathan
Vaish
Kalash
Sindhi
Hallaki
Bene
Lodi
Tharu
Kamsali
Vysya
Naidu
Mala
Velama
Bhil
Satnami
Kashmiri_Pandit
Meghawal
Madiga
Kurumba
Kharia
Santhal
Sahariya
Uygur
Hazara
Brahui
Balochi
Makrani
Burusho
Bedouin
YMNJ
TURJ
ASHJ
GRKJ
ITAJ
SYRJ
ALGJ
MORJ
TUNJ
LIBJ
DJEJ
IRQJ
IRNJ
GEOJ
Druze
Palestinian
