# Supplementary material for: The Genetics of Bene Israel from India Reveals Both Substantial Jewish and Indian Ancestry
Source: PLoS One. 2016 Mar 24;11(3):e0152056. doi: 10.1371/journal.pone.0152056 (PMC4806850; doi:10.1371/journal.pone.0152056)
Supplement: S5 Fig — Standard errors estimations are based on 10,000 bootstrapping of Bene Israel individuals (Materials and Methods). Except for Italian Jews in some cases, in all other cases, Georgian, Iraqi, Syrian or Iranian Jews showed significantly (<e-8, Wilcoxon test) higher IBD sharing with Bene Israel as compared to other Jewish populations. (PDF) [file pone.0152056.s005.pdf]

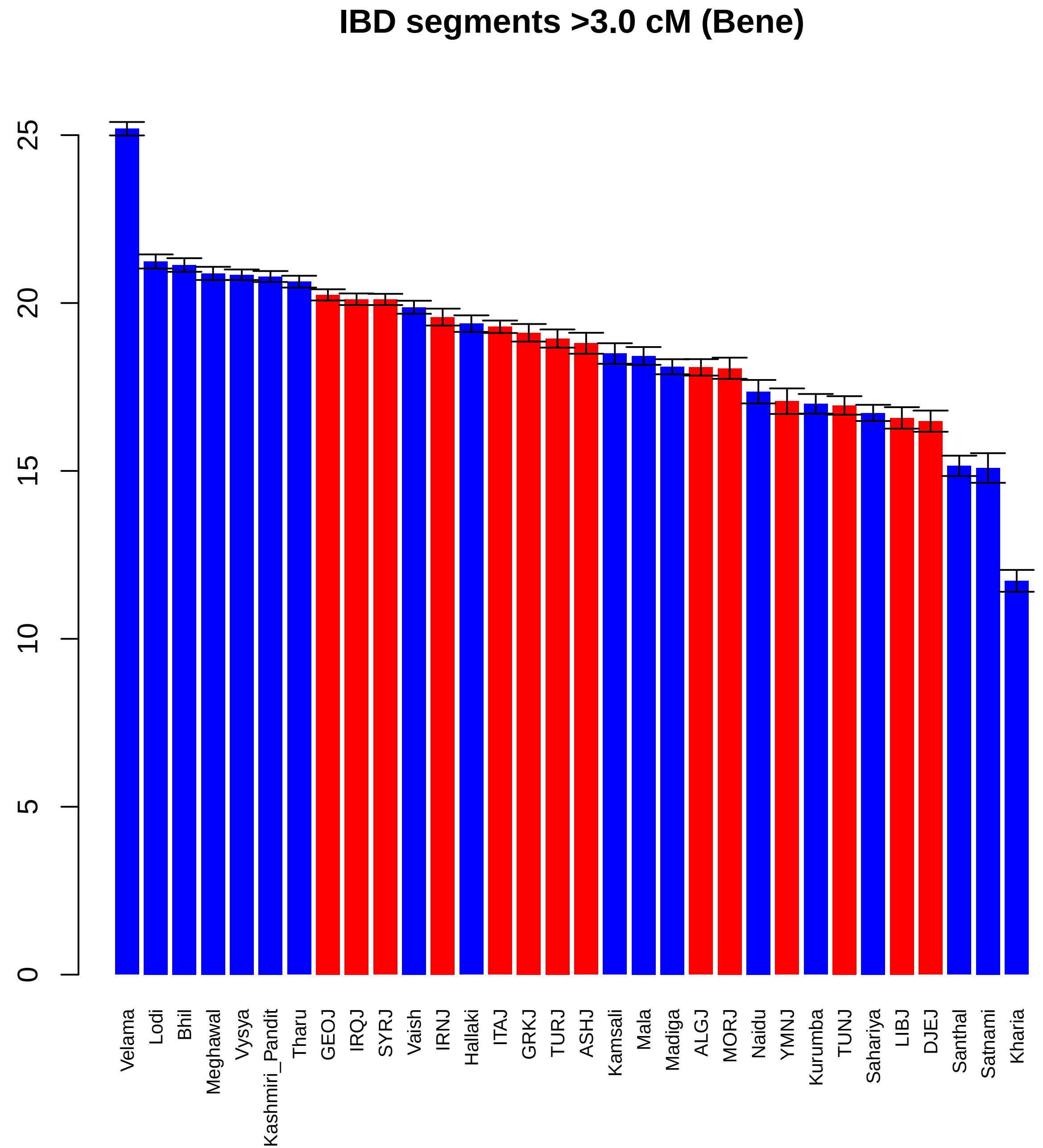

IBD segments >3.0 cM (Bene)
0
5
10
15
20
25
Velama
Lodi
Bhil
Meghawal
Vysya
Kashmiri_Pandit
Tharu
GEOJ
IRQJ
SYRJ
Vaish
IRNJ
Hallaki
ITAJ
GRKJ
TURJ
ASHJ
Kamsali
Mala
Madiga
ALGJ
MORJ
Naidu
YMNJ
Kurumba
TUNJ
Sahariya
LIBJ
DJEJ
Santhal
Satnami
Kharia

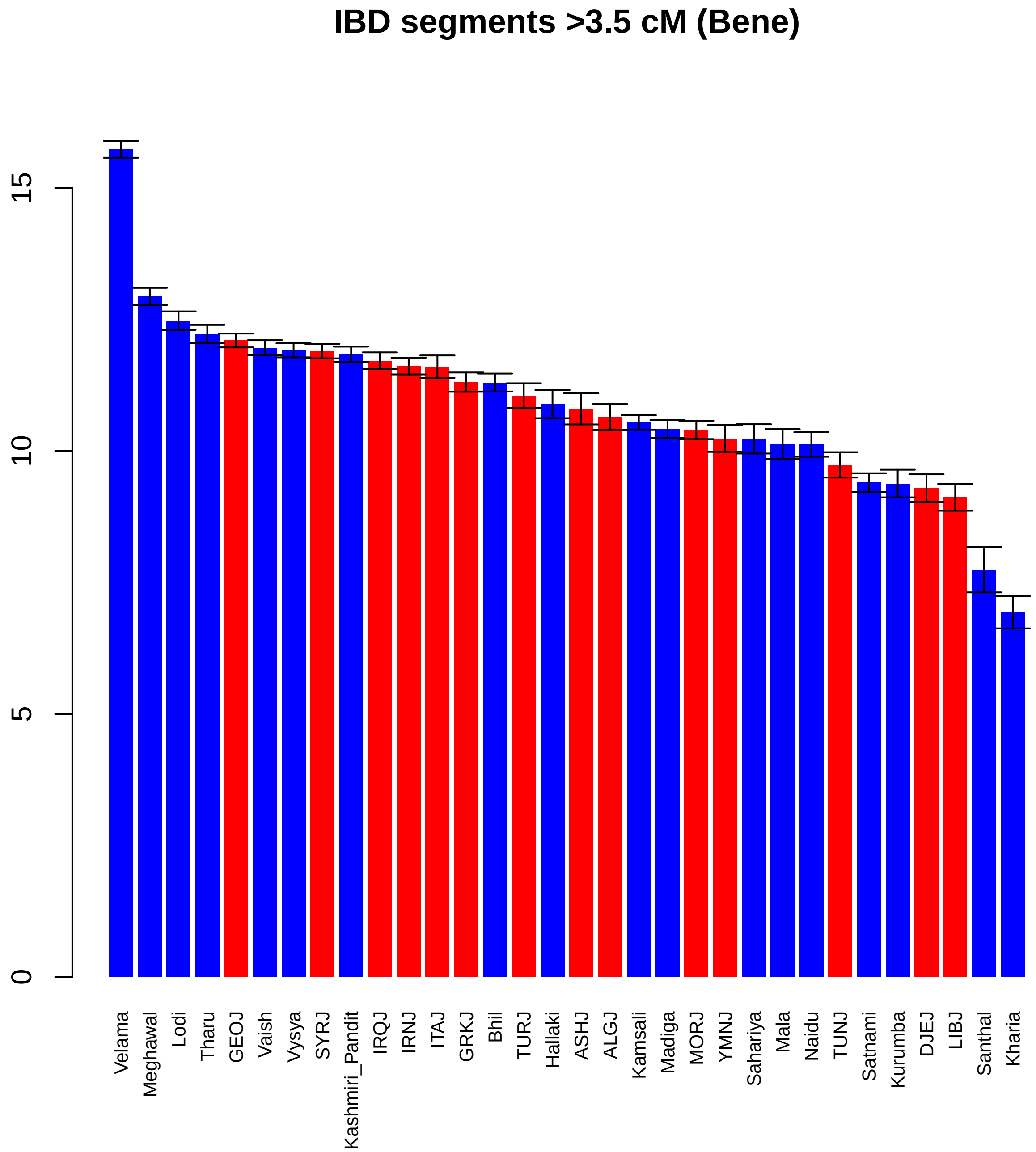
IBD segments >3.5 cM (Bene)
0
5
10
15
Velama
Meghawal
Lodi
Tharu
GEOJ
Vaish
Vysya
SYRJ
Kashmiri_Pandit
IRQJ
IRNJ
ITAJ
GRKJ
Bhil
TURJ
Hallaki
ASHJ
ALGJ
Kamsali
Madiga
MORJ
YMNJ
Sahariya
Mala
Naidu
TUNJ
Satnami
Kurumba
DJEJ
LIBJ
Santhal
Kharia

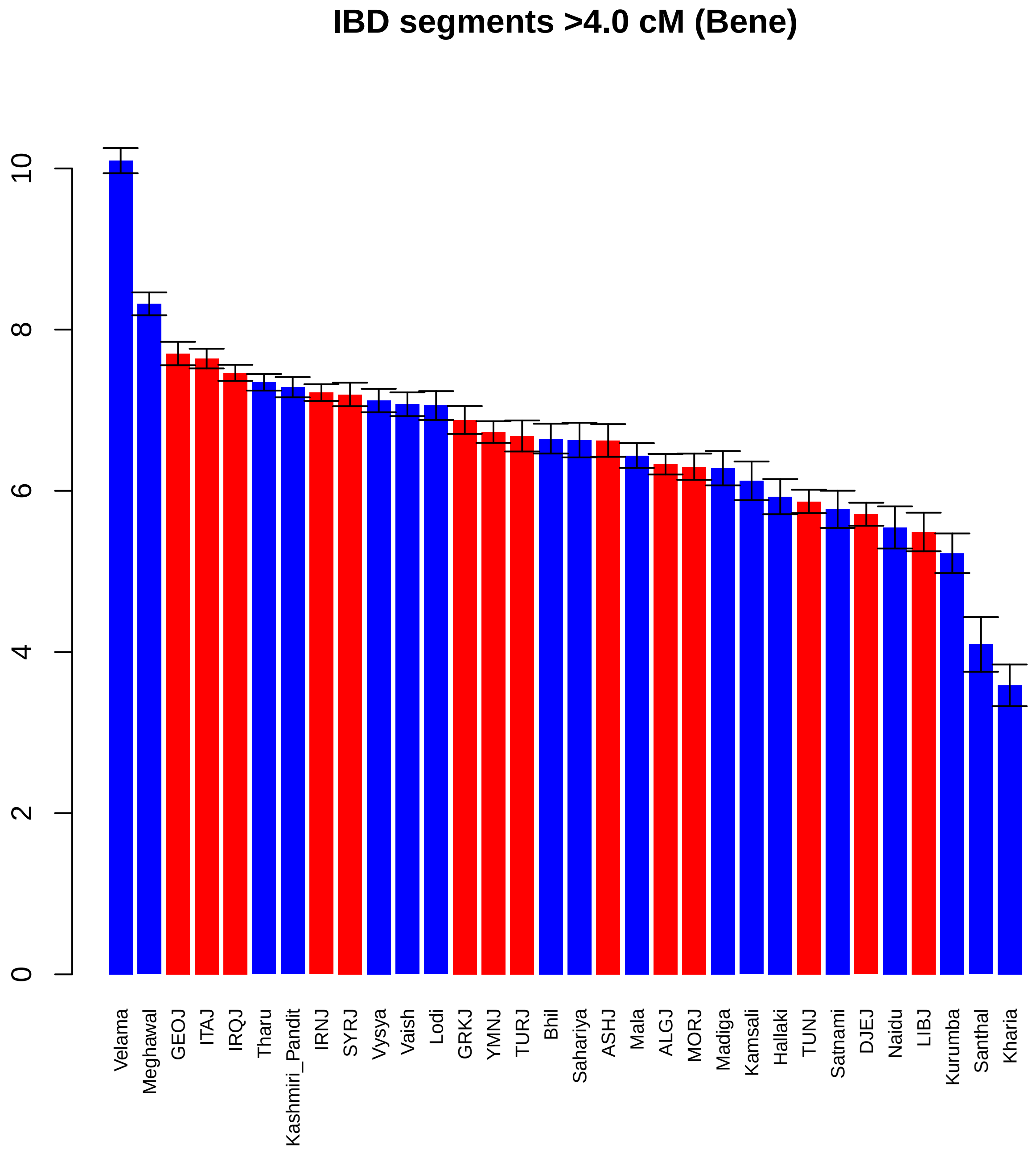
IBD segments >4.0 cM (Bene)
0
2
4
6
8
10
Velama
Meghawal
GEOJ
ITAJ
IRQJ
Tharu
Kashmiri_Pandit
IRNJ
SYRJ
Vysya
Vaish
Lodi
GRKJ
YMNJ
TURJ
Bhil
Sahariya
ASHJ
Mala
ALGJ
MORJ
Madiga
Kamsali
Hallaki
TUNJ
Satnami
DJEJ
Naidu
LIBJ
Kurumba
Santhal
Kharia

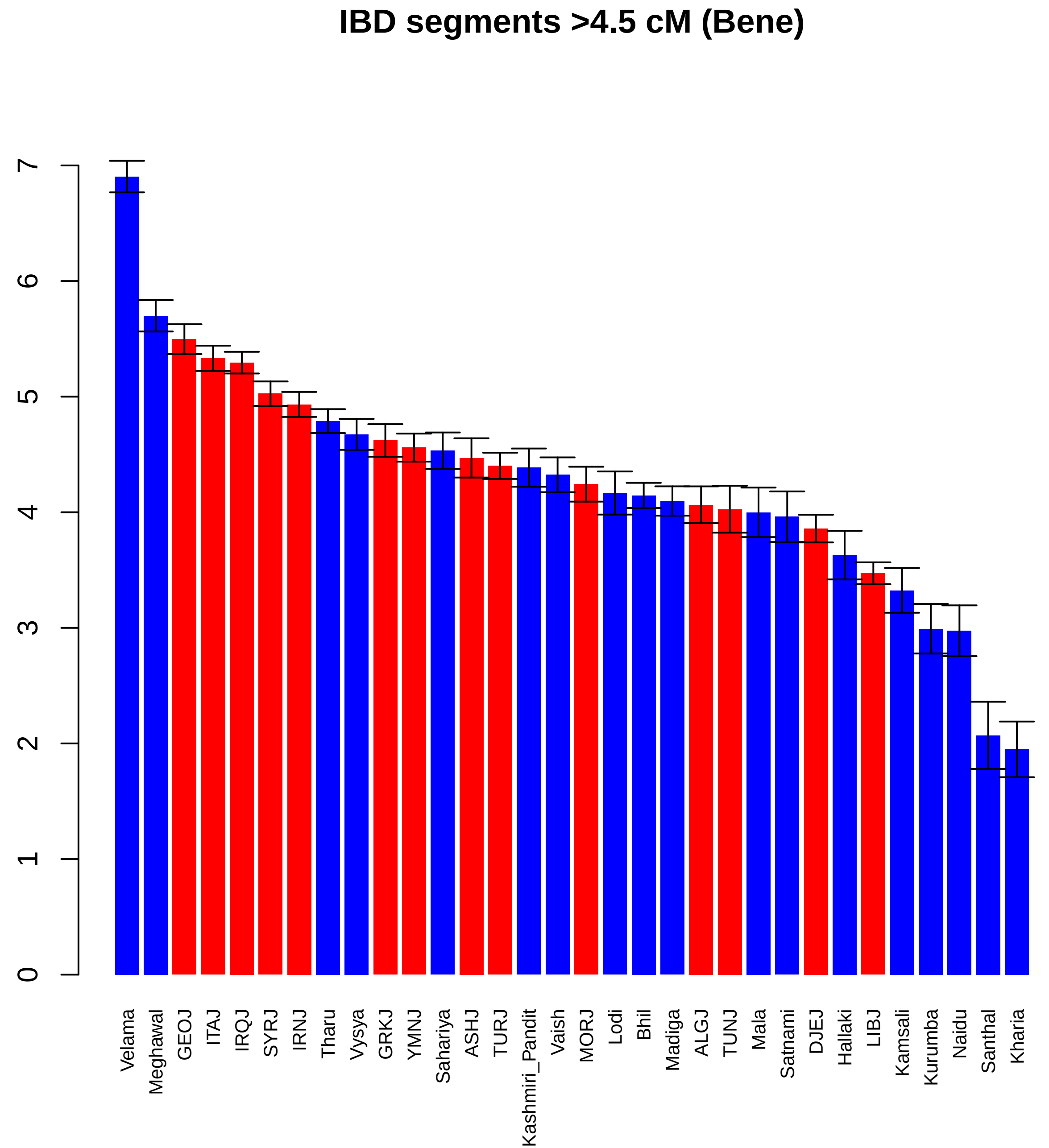

IBD segments >4.5 cM (Bene)
0
1
2
3
4
5
6
7
Velama
Meghawal
GEOJ
ITAJ
IRQJ
SYRJ
IRNJ
Tharu
Vysya
GRKJ
YMNJ
Sahariya
ASHJ
TURJ
Kashmiri_Pandit
Vaish
MORJ
Lodi
Bhil
Madiga
ALGJ
TUNJ
Mala
Satnami
DJEJ
Hallaki
LIBJ
Kamsali
Kurumba
Naidu
Santhal
Kharia

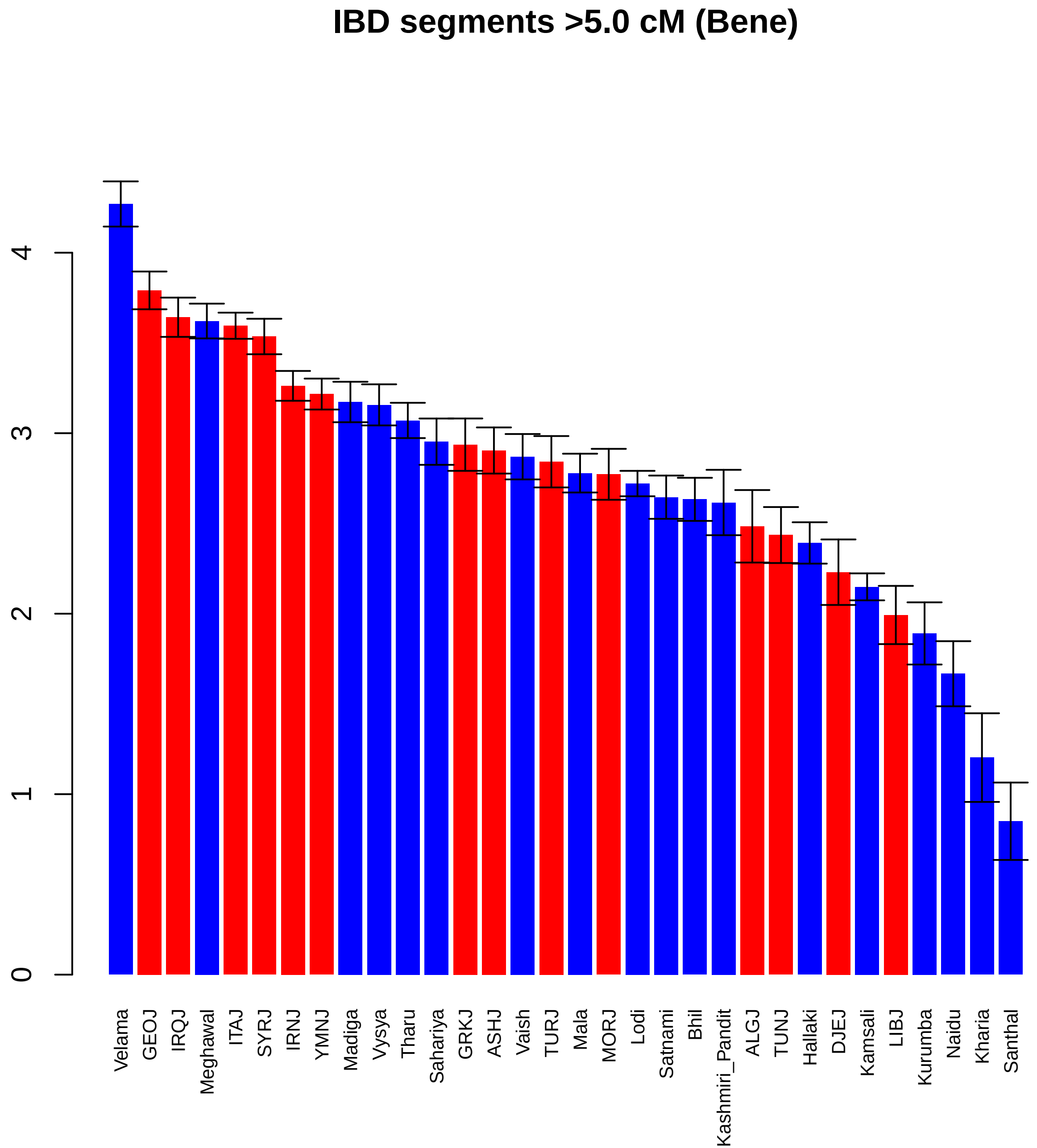

IBD segments >5.0 cM (Bene)
0
1
2
3
4
Velama
GEOJ
IRQJ
Meghawal
ITAJ
SYRJ
IRNJ
YMNJ
Madiga
Vysya
Tharu
Sahariya
GRKJ
ASHJ
Vaish
TURJ
Mala
MORJ
Lodi
Satnami
Bhil
Kashmiri_Pandit
ALGJ
TUNJ
Hallaki
DJEJ
Kamsali
LIBJ
Kurumba
Naidu
Kharia
Santhal
