# Supplementary material for: The Genetics of Bene Israel from India Reveals Both Substantial Jewish and Indian Ancestry
Source: PLoS One. 2016 Mar 24;11(3):e0152056. doi: 10.1371/journal.pone.0152056 (PMC4806850; doi:10.1371/journal.pone.0152056)
Supplement: S1 Table — (PDF) [file pone.0152056.s015.pdf]

Table S1. Samples and populations used in the current study and their annotations.

	Population	Population ID	No. of samples (before QC)	No. of samples (after QC)
**Jewish populations**	Bene Israel	Bene	19	18
	Algerian Jews	ALGJ	24	23
	Ashkenazi Jews	ASHJ	36	33
	Djerban Jews	DJEJ	18	9
	Georgian Jews	GEOJ	15	14
	Greek Jews	GRKJ	60	44
	Iranian Jews	IRNJ	118	20
	Iraqi Jews	IRQJ	40	22
	Italian Jews	ITAJ	28	15
	Libyan Jews	LIBJ	38	36
	Moroccan Jews	MORJ	38	33
	Syrian Jews	SYRJ	33	20
	Tunisian Jews	TUNJ	29	26
	Turkish Jews	TURJ	19	18
	Yemenite Jews	YMNJ	36	34
	**Total (Jewish populations)**		**551**	**365**
**Indian populations**	Bhil	Bhil	7	7
	Hallaki	Hallaki	7	7
	Kamsali	Kamsali	4	4
	Kashmiri Pandit	Kashmiri_Pandit	5	5
	Kharia	Kharia	6	6
	Kurumba	Kurumba	9	9
	Lodi	Lodi	5	5
	Madiga	Madiga	4	4
	Mala	Mala	3	3
	Meghawal	Meghawal	5	5
	Naidu	Naidu	4	4
	Sahariya	Sahariya	4	4
	Santhal	Santhal	7	7
	Satnami	Satnami	4	4
	Tharu	Tharu	9	9
	Vaish	Vaish	4	4
	Velama	Velama	4	4
	Vysya	Vysya	5	5
	**Total (Indian populations)**		**96**	**96**
**Pakistani populations**	Balochi		5	5

	Brahui	Brahui	5	5
	Makrani	Makrani	5	4
	Sindhi	Sindhi	5	4
	Pathan	Pathan	5	5
	Burusho	Burusho	5	5
	Hazara	Hazara	5	5
	Uygur	Uygur	5	5
	Kalash	Kalash	5	5
	Total (Pakistani populations)		45	43
**HapMap3 populations**	African ancestry in Southwest USA	ASW	47	47
	Utah residents with ancestry from northern and western Europe	CEU	110	97
	Han Chinese in Beijing, China	CHB	88	72
	Chinese in Metropolitan Denver, Colorado	CHD	86	75
	Gujarati Indians in Houston, Texas	GIH	85	77
	Japanese in Tokyo, Japan	JPT	87	78
	Luhya in Webuye, Kenya	LWK	78	74
	Mexican ancestry in Los Angeles, California	MEX	53	44
	Maasai in Kinyawa, Kenya	MKK	140	88
	Toscans in Italy	TSI	87	85
	Yoruba in Ibadan, Nigeria	YRI	110	107
	**Total (HapMap3 populations)**		**971**	**844**
**Middle Eastern populations**	Druze	Druze	5	5
	Bedouin	Bedouin	5	5
	Palestinians	Palestinians	5	5
	**Total (Middle Eastern populations)**		**15**	**15**
	**Total (all populations)**		**1678**	**1363**
