# Supplementary material for: The Genetics of Bene Israel from India Reveals Both Substantial Jewish and Indian Ancestry
Source: PLoS One. 2016 Mar 24;11(3):e0152056. doi: 10.1371/journal.pone.0152056 (PMC4806850; doi:10.1371/journal.pone.0152056)
Supplement: S3 Table — (PDF) [file pone.0152056.s017.pdf]

Table S3. ALDER admixture estimated time and proportions for Bene Israel, using different Jewish and Indian populations.

Indian population	Jewish population	P-value	Admixture time (generations), 2-reference populations	Admixture time (generations), 1-reference populations (Indian population)	Admixture time (generations), 1 reference populations (Jewish population)	Lower bound of admixture proportions (2-reference populations)
Bhil	ALGJ	3.70E-23	25.66 ± 2.46	23.76 ± 2.61	28.04 ± 3.64	23.08%
Bhil	ASHJ	1.20E-26	26.35 ± 2.36	23.76 ± 2.61	23.86 ± 3.09	21.95%
Bhil	DJEJ	3.60E-21	26.51 ± 2.42	23.76 ± 2.61	27.26 ± 5.32	18.33%
Bhil	GEOJ	5.50E-09	24.83 ± 3.72	23.76 ± 2.61	26.98 ± 2.35	23.29%
Bhil	GRKJ	1.90E-26	21.88 ± 1.97	23.76 ± 2.61	24.53 ± 2.86	21.20%
Bhil	IRNJ	2.40E-17	25.19 ± 2.77	23.76 ± 2.61	23.69 ± 2.99	16.52%
Bhil	IRQJ	3.70E-18	22.17 ± 2.21	23.76 ± 2.61	22.60 ± 3.80	18.25%
Bhil	ITAJ	9.80E-22	22.64 ± 2.24	23.76 ± 2.61	24.01 ± 3.57	17.45%
Bhil	LIBJ	9.40E-19	24.67 ± 2.62	23.76 ± 2.61	24.01 ± 4.41	16.56%
Bhil	MORJ	4.90E-20	26.21 ± 2.69	23.76 ± 2.61	24.66 ± 3.16	21.77%
Bhil	SYRJ	9.60E-38	24.60 ± 1.86	23.76 ± 2.61	24.33 ± 4.15	22.74%
Bhil	TUNJ	4.60E-18	24.79 ± 2.68	23.76 ± 2.61	23.82 ± 4.78	19.75%
Bhil	TURJ	1.40E-11	24.31 ± 3.24	23.76 ± 2.61	25.06 ± 4.00	24.52%
Bhil	YMNJ	9.50E-17	25.13 ± 2.81	23.76 ± 2.61	27.12 ± 3.67	19.41%
Hallaki	ALGJ	1.20E-18	25.93 ± 2.38	21.97 ± 2.41	28.04 ± 3.64	20.80%
Hallaki	ASHJ	2.30E-14	25.24 ± 3.04	21.97 ± 2.41	23.86 ± 3.09	17.64%
Hallaki	DJEJ	6.50E-13	24.04 ± 3.04	21.97 ± 2.41	27.26 ± 5.32	15.09%
Hallaki	GEOJ	4.20E-15	22.77 ± 2.66	21.97 ± 2.41	26.98 ± 2.35	19.87%
Hallaki	GRKJ	4.20E-15	21.73 ± 2.30	21.97 ± 2.41	24.53 ± 2.86	19.18%
Hallaki	IRNJ	4.80E-14	23.21 ± 2.82	21.97 ± 2.41	23.69 ± 2.99	14.31%
Hallaki	IRQJ	9.30E-16	21.37 ± 1.41	21.97 ± 2.41	22.60 ± 3.80	15.47%
Hallaki	ITAJ	6.70E-11	22.45 ± 3.08	21.97 ± 2.41	24.01 ± 3.57	15.77%
Hallaki	LIBJ	1.40E-13	24.10 ± 2.98	21.97 ± 2.41	24.01 ± 4.41	13.96%
Hallaki	MORJ	5.80E-14	24.81 ± 2.49	21.97 ± 2.41	24.66 ± 3.16	17.52%
Hallaki	SYRJ	5.40E-10	24.17 ± 3.45	21.97 ± 2.41	24.33 ± 4.15	20.63%
Hallaki	TUNJ	1.10E-13	24.92 ± 3.07	21.97 ± 2.41	23.82 ± 4.78	17.40%
Hallaki	TURJ	1.40E-11	23.81 ± 3.08	21.97 ± 2.41	25.06 ± 4.00	20.63%
Hallaki	YMNJ	1.90E-19	24.03 ± 2.50	21.97 ± 2.41	27.12 ± 3.67	16.56%
Kamsali	ASHJ	1.20E-11	25.41 ± 3.38	19.86 ± 2.52	23.86 ± 3.09	17.73%
Kamsali	GRKJ	2.50E-12	20.83 ± 2.69	19.86 ± 2.52	24.53 ± 2.86	18.19%
Kamsali	IRNJ	3.00E-15	23.60 ± 2.76	19.86 ± 2.52	23.69 ± 2.99	15.46%
Kamsali	IRQJ	3.10E-08	22.44 ± 3.14	19.86 ± 2.52	22.60 ± 3.80	17.29%
Kamsali	ITAJ	4.20E-15	21.41 ± 2.52	19.86 ± 2.52	24.01 ± 3.57	14.21%
Kamsali	LIBJ	1.10E-14	25.01 ± 2.73	19.86 ± 2.52	24.01 ± 4.41	15.33%
Kamsali	MORJ	1.20E-18	25.20 ± 2.65	19.86 ± 2.52	24.66 ± 3.16	18.32%
Kamsali	SYRJ	2.20E-15	23.96 ± 2.33	19.86 ± 2.52	24.33 ± 4.15	19.98%
Kamsali	TUNJ	5.00E-10	24.04 ± 2.44	19.86 ± 2.52	23.82 ± 4.78	17.11%

Kamsali	TURJ	1.10E-13	25.07 ± 3.09	19.86 ± 2.52	25.06 ± 4.00	22.36%
Kharia	GRKJ	1.40E-17	25.98 ± 2.84	25.16 ± 2.28	24.53 ± 2.86	11.93%
Kharia	IRQJ	1.20E-11	25.95 ± 3.45	25.16 ± 2.28	22.60 ± 3.80	9.93%
Kharia	ITAJ	1.30E-16	28.25 ± 3.17	25.16 ± 2.28	24.01 ± 3.57	11.31%
Kharia	MORJ	6.90E-17	31.53 ± 3.51	25.16 ± 2.28	24.66 ± 3.16	12.93%
Kharia	SYRJ	2.50E-23	30.27 ± 2.84	25.16 ± 2.28	24.33 ± 4.15	13.23%
Kharia	TUNJ	7.60E-15	29.37 ± 3.48	25.16 ± 2.28	23.82 ± 4.78	11.87%
Kharia	TURJ	6.00E-19	31.14 ± 3.29	25.16 ± 2.28	25.06 ± 4.00	14.40%
Kharia	YMNJ	5.10E-14	30.99 ± 3.77	25.16 ± 2.28	27.12 ± 3.67	12.94%
Kurumba	GRKJ	2.70E-17	22.84 ± 2.52	20.95 ± 2.41	24.53 ± 2.86	20.35%
Kurumba	IRNJ	2.40E-11	26.01 ± 3.50	20.95 ± 2.41	23.69 ± 2.99	16.09%
Kurumba	IRQJ	8.80E-12	23.37 ± 3.09	20.95 ± 2.41	22.60 ± 3.80	17.56%
Kurumba	ITAJ	1.50E-13	23.70 ± 2.93	20.95 ± 2.41	24.01 ± 3.57	16.21%
Kurumba	LIBJ	3.20E-11	26.09 ± 3.53	20.95 ± 2.41	24.01 ± 4.41	15.51%
Kurumba	SYRJ	9.80E-21	25.55 ± 2.58	20.95 ± 2.41	24.33 ± 4.15	20.67%
Kurumba	TUNJ	2.50E-11	26.62 ± 3.58	20.95 ± 2.41	23.82 ± 4.78	18.99%
Kurumba	TURJ	1.40E-12	24.26 ± 3.11	20.95 ± 2.41	25.06 ± 4.00	21.53%
Lodi	GRKJ	5.20E-19	26.24 ± 2.76	20.58 ± 2.61	24.53 ± 2.86	27.31%
Lodi	IRQJ	1.00E-06	25.01 ± 3.24	20.58 ± 2.61	22.60 ± 3.80	20.76%
Madiga	GRKJ	4.70E-08	25.29 ± 3.98	22.01 ± 2.58	24.53 ± 2.86	20.62%
Madiga	IRQJ	1.10E-05	27.50 ± 5.04	22.01 ± 2.58	22.60 ± 3.80	20.58%
Madiga	ITAJ	1.40E-05	26.83 ± 4.96	22.01 ± 2.58	24.01 ± 3.57	17.57%
Madiga	MORJ	1.20E-07	27.94 ± 4.49	22.01 ± 2.58	24.66 ± 3.16	19.72%
Mala	ASHJ	4.90E-10	28.65 ± 4.08	26.35 ± 2.31	23.86 ± 3.09	19.16%
Mala	GEOJ	1.30E-08	32.52 ± 4.97	26.35 ± 2.31	26.98 ± 2.35	29.83%
Mala	GRKJ	6.20E-09	24.00 ± 3.60	26.35 ± 2.31	24.53 ± 2.86	18.80%
Mala	IRNJ	7.10E-08	30.03 ± 4.77	26.35 ± 2.31	23.69 ± 2.99	17.69%
Mala	IRQJ	2.60E-07	26.22 ± 4.31	26.35 ± 2.31	22.60 ± 3.80	18.58%
Mala	ITAJ	7.20E-08	28.74 ± 4.57	26.35 ± 2.31	24.01 ± 3.57	18.37%
Mala	MORJ	9.30E-08	28.68 ± 4.59	26.35 ± 2.31	24.66 ± 3.16	19.90%
Mala	SYRJ	4.10E-08	29.81 ± 4.67	26.35 ± 2.31	24.33 ± 4.15	24.20%
Mala	TUNJ	4.50E-12	29.07 ± 3.80	26.35 ± 2.31	23.82 ± 4.78	19.48%
Mala	YMNJ	3.50E-09	31.03 ± 4.60	26.35 ± 2.31	27.12 ± 3.67	21.71%
Meghawal	GRKJ	1.50E-06	29.14 ± 5.02	23.59 ± 3.28	24.53 ± 2.86	NA
Meghawal	IRQJ	0.00024	26.34 ± 5.39	23.59 ± 3.28	22.60 ± 3.80	26.50%
Meghawal	TURJ	6.00E-05	29.90 ± 5.81	23.59 ± 3.28	25.06 ± 4.00	NA
Naidu	ASHJ	8.60E-10	20.93 ± 3.01	20.72 ± 3.51	23.86 ± 3.09	17.99%
Naidu	GRKJ	2.00E-08	19.43 ± 3.00	20.72 ± 3.51	24.53 ± 2.86	21.89%
Naidu	IRNJ	0.00047	23.39 ± 4.93	20.72 ± 3.51	23.69 ± 2.99	17.00%
Naidu	IRQJ	8.00E-07	19.66 ± 3.33	20.72 ± 3.51	22.60 ± 3.80	18.60%
Naidu	ITAJ	1.30E-09	20.05 ± 2.91	20.72 ± 3.51	24.01 ± 3.57	16.87%
Naidu	LIBJ	2.80E-08	23.44 ± 3.64	20.72 ± 3.51	24.01 ± 4.41	16.72%
Naidu	MORJ	8.90E-07	22.56 ± 3.83	20.72 ± 3.51	24.66 ± 3.16	19.60%
Naidu	SYRJ	2.90E-12	20.95 ± 2.72	20.72 ± 3.51	24.33 ± 4.15	22.35%
Naidu	TUNJ	2.80E-07	22.71 ± 3.74	20.72 ± 3.51	23.82 ± 4.78	19.17%
Naidu	TURJ	3.50E-08	21.63 ± 3.38	20.72 ± 3.51	25.06 ± 4.00	25.58%
Sahariya	ALGJ	7.90E-15	28.86 ± 3.42	24.24 ± 2.70	28.04 ± 3.64	17.19%
Sahariya	ASHJ	1.30E-19	25.39 ± 2.56	24.24 ± 2.70	23.86 ± 3.09	13.72%

Sahariya	DJEJ	8.40E-11	27.77 ± 3.82	24.24 ± 2.70	27.26 ± 5.32	13.09%
Sahariya	GEOJ	1.20E-08	27.21 ± 4.15	24.24 ± 2.70	26.98 ± 2.35	16.64%
Sahariya	GRKJ	3.10E-14	22.29 ± 2.69	24.24 ± 2.70	24.53 ± 2.86	14.05%
Sahariya	IRNJ	1.90E-08	25.82 ± 3.97	24.24 ± 2.70	23.69 ± 2.99	11.71%
Sahariya	IRQJ	8.20E-09	24.06 ± 2.95	24.24 ± 2.70	22.60 ± 3.80	13.01%
Sahariya	ITAJ	8.60E-12	24.21 ± 3.20	24.24 ± 2.70	24.01 ± 3.57	12.44%
Sahariya	LIBJ	3.90E-12	26.09 ± 3.40	24.24 ± 2.70	24.01 ± 4.41	12.39%
Sahariya	MORJ	8.80E-16	25.30 ± 2.91	24.24 ± 2.70	24.66 ± 3.16	13.75%
Sahariya	SYRJ	8.00E-22	25.40 ± 2.50	24.24 ± 2.70	24.33 ± 4.15	15.44%
Sahariya	TUNJ	1.80E-10	24.90 ± 3.47	24.24 ± 2.70	23.82 ± 4.78	13.08%
Sahariya	TURJ	7.50E-09	25.92 ± 3.91	24.24 ± 2.70	25.06 ± 4.00	17.84%
Sahariya	YMNJ	5.10E-10	26.52 ± 3.78	24.24 ± 2.70	27.12 ± 3.67	14.55%
Santhal	ASHJ	8.70E-15	28.41 ± 3.37	24.14 ± 3.06	23.86 ± 3.09	15.01%
Santhal	DJEJ	2.20E-15	29.25 ± 3.41	24.14 ± 3.06	27.26 ± 5.32	13.97%
Santhal	GRKJ	7.60E-15	25.76 ± 3.05	24.14 ± 3.06	24.53 ± 2.86	15.24%
Santhal	IRNJ	4.10E-13	29.57 ± 3.72	24.14 ± 3.06	23.69 ± 2.99	13.80%
Santhal	IRQJ	8.50E-13	25.60 ± 3.25	24.14 ± 3.06	22.60 ± 3.80	13.40%
Santhal	ITAJ	8.30E-12	26.69 ± 3.52	24.14 ± 3.06	24.01 ± 3.57	13.43%
Santhal	LIBJ	3.50E-11	29.67 ± 4.02	24.14 ± 3.06	24.01 ± 4.41	13.98%
Santhal	MORJ	7.80E-19	28.71 ± 3.04	24.14 ± 3.06	24.66 ± 3.16	15.11%
Santhal	SYRJ	4.10E-17	28.87 ± 3.20	24.14 ± 3.06	24.33 ± 4.15	16.48%
Santhal	TUNJ	5.50E-13	27.54 ± 3.48	24.14 ± 3.06	23.82 ± 4.78	14.21%
Santhal	TURJ	5.50E-12	29.67 ± 3.89	24.14 ± 3.06	25.06 ± 4.00	18.19%
Santhal	YMNJ	1.00E-16	28.52 ± 3.20	24.14 ± 3.06	27.12 ± 3.67	15.08%
Satnami	ASHJ	1.90E-11	24.49 ± 3.28	20.05 ± 2.94	23.86 ± 3.09	19.13%
Satnami	GRKJ	1.60E-17	20.14 ± 2.21	20.05 ± 2.94	24.53 ± 2.86	18.49%
Satnami	IRNJ	5.80E-11	23.07 ± 3.15	20.05 ± 2.94	23.69 ± 2.99	14.75%
Satnami	IRQJ	6.60E-18	19.14 ± 2.07	20.05 ± 2.94	22.60 ± 3.80	14.63%
Satnami	ITAJ	2.00E-11	22.63 ± 3.03	20.05 ± 2.94	24.01 ± 3.57	17.07%
Satnami	LIBJ	5.90E-15	24.32 ± 2.87	20.05 ± 2.94	24.01 ± 4.41	15.60%
Satnami	MORJ	4.80E-15	23.13 ± 2.72	20.05 ± 2.94	24.66 ± 3.16	18.04%
Satnami	SYRJ	1.20E-12	22.99 ± 2.94	20.05 ± 2.94	24.33 ± 4.15	19.69%
Satnami	TUNJ	4.60E-09	23.42 ± 3.49	20.05 ± 2.94	23.82 ± 4.78	17.34%
Satnami	TURJ	1.10E-09	23.10 ± 3.34	20.05 ± 2.94	25.06 ± 4.00	22.21%
Tharu	GRKJ	1.10E-25	22.96 ± 2.09	20.66 ± 2.14	24.53 ± 2.86	32.20%
Tharu	IRNJ	1.30E-21	25.20 ± 2.49	20.66 ± 2.14	23.69 ± 2.99	20.73%
Tharu	IRQJ	1.90E-14	22.40 ± 1.92	20.66 ± 2.14	22.60 ± 3.80	24.33%
Tharu	ITAJ	2.20E-33	23.95 ± 1.92	20.66 ± 2.14	24.01 ± 3.57	22.58%
Tharu	LIBJ	8.50E-20	25.42 ± 2.63	20.66 ± 2.14	24.01 ± 4.41	21.27%
Tharu	SYRJ	1.30E-27	24.83 ± 1.75	20.66 ± 2.14	24.33 ± 4.15	32.34%
Tharu	TUNJ	1.50E-20	24.85 ± 2.52	20.66 ± 2.14	23.82 ± 4.78	26.35%
Tharu	TURJ	2.20E-25	24.23 ± 2.22	20.66 ± 2.14	25.06 ± 4.00	37.03%
Vaish	GRKJ	2.80E-08	24.01 ± 3.73	23.53 ± 3.10	24.53 ± 2.86	NA
Vaish	IRNJ	5.50E-05	29.53 ± 5.71	23.53 ± 3.10	23.69 ± 2.99	30.73%
Vaish	IRQJ	4.00E-06	22.99 ± 3.89	23.53 ± 3.10	22.60 ± 3.80	32.77%
Vaish	ITAJ	0.002	24.02 ± 5.40	23.53 ± 3.10	24.01 ± 3.57	31.69%
Vaish	MORJ	2.10E-09	29.60 ± 4.34	23.53 ± 3.10	24.66 ± 3.16	NA
Vaish	SYRJ	4.10E-08	29.29 ± 4.59	23.53 ± 3.10	24.33 ± 4.15	NA

Velama	GRKJ	2.60E-09	29.00 ± 3.81	23.31 ± 2.43	24.53 ± 2.86	26.75%
Vysya	ALGJ	6.60E-13	26.16 ± 3.31	22.49 ± 3.16	28.04 ± 3.64	14.49%
Vysya	ASHJ	1.90E-10	27.36 ± 3.34	22.49 ± 3.16	23.86 ± 3.09	15.30%
Vysya	GEOJ	2.30E-15	25.28 ± 2.91	22.49 ± 3.16	26.98 ± 2.35	15.39%
Vysya	GRKJ	4.20E-17	23.56 ± 2.61	22.49 ± 3.16	24.53 ± 2.86	15.43%
Vysya	IRNJ	5.00E-11	24.20 ± 2.40	22.49 ± 3.16	23.69 ± 2.99	11.79%
Vysya	IRQJ	8.60E-07	23.53 ± 2.87	22.49 ± 3.16	22.60 ± 3.80	13.65%
Vysya	ITAJ	6.00E-14	21.83 ± 2.67	22.49 ± 3.16	24.01 ± 3.57	12.06%
Vysya	LIBJ	4.10E-07	26.70 ± 3.42	22.49 ± 3.16	24.01 ± 4.41	13.08%
Vysya	MORJ	9.70E-13	28.18 ± 3.17	22.49 ± 3.16	24.66 ± 3.16	15.14%
Vysya	SYRJ	1.20E-19	25.93 ± 2.44	22.49 ± 3.16	24.33 ± 4.15	16.34%
Vysya	TUNJ	2.10E-09	26.05 ± 2.74	22.49 ± 3.16	23.82 ± 4.78	14.14%
Vysya	TURJ	4.10E-17	24.53 ± 2.70	22.49 ± 3.16	25.06 ± 4.00	16.83%
Vysya	YMNJ	1.10E-07	26.35 ± 3.75	22.49 ± 3.16	27.12 ± 3.67	14.15%

The P-value given by ALDER is corrected for multiple testing. ALDER estimations for ALDER estimations of admixture proportions using 1-reference population are presented in Table 1 and the estimations here are based on 2-reference populations (see also SI Appendix, Materials and Methods). 'NA' is given in the few cases the solution for the admixture proportion was not defined.
