# Supplementary material for: The Genetics of Bene Israel from India Reveals Both Substantial Jewish and Indian Ancestry
Source: PLoS One. 2016 Mar 24;11(3):e0152056. doi: 10.1371/journal.pone.0152056 (PMC4806850; doi:10.1371/journal.pone.0152056)
Supplement: S4 Table — (PDF) [file pone.0152056.s018.pdf]

Table S4. ALDER admixture estimated time and proportions for Bene Israel, using different Jewish/Middle Eastern and Indian populations.

Indian population	Jewish / Middle Eastern population	P-value	Admixture time (generations), 2-reference populations	Admixture time (generations), 1-reference populations (Indian population)	Admixture time (generations), 1 reference populations (Jewish population)	Lower bound of admixture proportions (2-reference populations)
Bhil	ALGJ	1.90E-25	26.68 ± 2.33	23.92 ± 2.30	28.01 ± 3.72	24.05%
Bhil	ASHJ	9.70E-25	27.02 ± 2.50	23.92 ± 2.30	23.59 ± 2.88	22.59%
Bhil	DJEJ	1.60E-11	27.10 ± 2.99	23.92 ± 2.30	28.12 ± 4.87	18.93%
Bhil	GEOJ	1.50E-07	25.07 ± 4.04	23.92 ± 2.30	26.55 ± 2.22	23.48%
Bhil	GRKJ	6.10E-33	22.86 ± 1.70	23.92 ± 2.30	23.54 ± 2.82	22.49%
Bhil	IRNJ	3.20E-16	25.30 ± 2.87	23.92 ± 2.30	23.05 ± 3.35	16.48%
Bhil	IRQJ	8.80E-17	22.93 ± 2.47	23.92 ± 2.30	22.95 ± 4.19	18.94%
Bhil	ITAJ	5.20E-24	23.66 ± 2.22	23.92 ± 2.30	23.76 ± 3.44	17.87%
Bhil	LIBJ	1.60E-22	25.32 ± 2.45	23.92 ± 2.30	23.10 ± 4.10	16.58%
Bhil	MORJ	2.80E-27	26.99 ± 2.38	23.92 ± 2.30	24.60 ± 3.17	22.60%
Bhil	SYRJ	2.80E-30	25.68 ± 1.99	23.92 ± 2.30	24.44 ± 4.52	23.29%
Bhil	TUNJ	1.20E-23	25.34 ± 2.40	23.92 ± 2.30	24.72 ± 4.35	20.35%
Bhil	TURJ	7.00E-16	24.76 ± 2.84	23.92 ± 2.30	24.06 ± 4.08	24.55%
Bhil	YMNJ	1.60E-17	26.84 ± 2.93	23.92 ± 2.30	28.20 ± 4.32	20.11%
Bhil	Druze	5.40E-12	25.28 ± 2.81	23.92 ± 2.30	29.67 ± 4.81	14.88%
Bhil	Bedouin	0.0021	24.57 ± 5.49	23.92 ± 2.30	27.48 ± 7.74	11.62%
Bhil	Palestinian	3.70E-09	26.52 ± 3.77	23.92 ± 2.30	30.32 ± 6.70	19.65%
Hallaki	ALGJ	4.70E-10	27.46 ± 3.52	21.86 ± 2.74	28.01 ± 3.72	21.87%
Hallaki	ASHJ	8.20E-12	26.58 ± 3.50	21.86 ± 2.74	23.59 ± 2.88	18.97%
Hallaki	GEOJ	4.30E-14	23.29 ± 2.82	21.86 ± 2.74	26.55 ± 2.22	20.82%
Hallaki	GRKJ	4.90E-12	22.34 ± 2.56	21.86 ± 2.74	23.54 ± 2.82	19.96%
Hallaki	IRNJ	2.30E-09	24.33 ± 3.56	21.86 ± 2.74	23.05 ± 3.35	15.19%
Hallaki	IRQJ	3.10E-14	22.35 ± 2.13	21.86 ± 2.74	22.95 ± 4.19	15.92%
Hallaki	ITAJ	3.70E-09	24.35 ± 3.60	21.86 ± 2.74	23.76 ± 3.44	16.88%
Hallaki	LIBJ	1.60E-08	24.86 ± 3.80	21.86 ± 2.74	23.10 ± 4.10	14.16%
Hallaki	MORJ	1.10E-10	25.82 ± 3.10	21.86 ± 2.74	24.60 ± 3.17	18.37%
Hallaki	SYRJ	1.90E-07	26.14 ± 4.24	21.86 ± 2.74	24.44 ± 4.52	21.68%
Hallaki	TUNJ	3.20E-10	26.25 ± 3.69	21.86 ± 2.74	24.72 ± 4.35	18.21%
Hallaki	TURJ	4.20E-08	25.63 ± 3.87	21.86 ± 2.74	24.06 ± 4.08	22.08%
Hallaki	Bedouin	6.50E-05	24.58 ± 4.75	21.86 ± 2.74	27.48 ± 7.74	11.11%
Kamsali	GRKJ	2.90E-10	20.17 ± 2.81	19.95 ± 2.42	23.54 ± 2.82	18.68%
Kamsali	IRNJ	1.80E-11	25.28 ± 3.37	19.95 ± 2.42	23.05 ± 3.35	17.53%
Kamsali	IRQJ	6.70E-06	21.71 ± 3.35	19.95 ± 2.42	22.95 ± 4.19	17.03%
Kamsali	ITAJ	7.80E-10	22.08 ± 2.94	19.95 ± 2.42	23.76 ± 3.44	14.78%
Kamsali	SYRJ	2.70E-09	24.13 ± 2.69	19.95 ± 2.42	24.44 ± 4.52	19.94%
Kamsali	TUNJ	2.70E-09	25.48 ± 2.36	19.95 ± 2.42	24.72 ± 4.35	19.25%
Kamsali	TURJ	1.60E-10	24.60 ± 3.41	19.95 ± 2.42	24.06 ± 4.08	21.56%
Kharia	GRKJ	7.50E-18	26.38 ± 2.86	24.91 ± 2.60	23.54 ± 2.82	12.19%

Kharia	IRQJ	6.80E-08	26.93 ± 4.25	24.91 ± 2.60	22.95 ± 4.19	10.26%
Kharia	ITAJ	1.40E-15	28.09 ± 3.25	24.91 ± 2.60	23.76 ± 3.44	11.11%
Kharia	SYRJ	6.50E-20	31.41 ± 2.75	24.91 ± 2.60	24.44 ± 4.52	13.34%
Kharia	TURJ	7.60E-19	30.16 ± 3.18	24.91 ± 2.60	24.06 ± 4.08	13.43%
Kurumba	GRKJ	8.40E-23	23.36 ± 2.25	20.72 ± 2.39	23.54 ± 2.82	21.58%
Kurumba	IRQJ	5.10E-13	23.40 ± 2.90	20.72 ± 2.39	22.95 ± 4.19	17.66%
Kurumba	ITAJ	2.10E-14	24.67 ± 2.96	20.72 ± 2.39	23.76 ± 3.44	17.08%
Kurumba	TURJ	7.60E-14	24.51 ± 3.00	20.72 ± 2.39	24.06 ± 4.08	22.24%
Lodi	IRQJ	1.70E-05	24.33 ± 3.89	20.13 ± 2.97	22.95 ± 4.19	19.33%
Madiga	GRKJ	4.90E-05	25.76 ± 4.93	22.68 ± 2.70	23.54 ± 2.82	20.47%
Madiga	IRQJ	0.00038	26.88 ± 5.57	22.68 ± 2.70	22.95 ± 4.19	18.82%
Madiga	ITAJ	0.0046	27.00 ± 6.27	22.68 ± 2.70	23.76 ± 3.44	16.81%
Madiga	MORJ	8.90E-06	28.64 ± 5.18	22.68 ± 2.70	24.60 ± 3.17	19.05%
Mala	ASHJ	7.90E-07	30.09 ± 5.07	28.03 ± 2.57	23.59 ± 2.88	21.93%
Mala	GRKJ	2.40E-06	25.41 ± 4.41	28.03 ± 2.57	23.54 ± 2.82	21.13%
Mala	IRQJ	7.20E-06	26.96 ± 4.84	28.03 ± 2.57	22.95 ± 4.19	20.00%
Mala	ITAJ	4.40E-05	30.54 ± 5.83	28.03 ± 2.57	23.76 ± 3.44	20.42%
Mala	MORJ	1.30E-06	30.11 ± 5.14	28.03 ± 2.57	24.60 ± 3.17	22.08%
Mala	Druze	4.30E-06	33.12 ± 5.86	28.03 ± 2.57	29.67 ± 4.81	23.17%
Meghawal	IRQJ	0.00083	27.65 ± 5.92	25.13 ± 4.39	22.95 ± 4.19	28.75%
Naidu	ASHJ	2.50E-05	20.70 ± 3.87	20.34 ± 3.71	23.59 ± 2.88	18.90%
Naidu	GRKJ	0.00025	19.46 ± 3.96	20.34 ± 3.71	23.54 ± 2.82	22.86%
Naidu	IRQJ	0.0026	19.35 ± 4.37	20.34 ± 3.71	22.95 ± 4.19	18.63%
Naidu	ITAJ	2.10E-05	20.70 ± 3.85	20.34 ± 3.71	23.76 ± 3.44	17.85%
Naidu	LIBJ	2.20E-05	24.41 ± 4.55	20.34 ± 3.71	23.10 ± 4.10	17.58%
Naidu	MORJ	0.00059	23.06 ± 4.86	20.34 ± 3.71	24.60 ± 3.17	20.75%
Naidu	SYRJ	3.00E-06	20.98 ± 3.67	20.34 ± 3.71	24.44 ± 4.52	22.02%
Naidu	TUNJ	0.00041	25.78 ± 5.36	20.34 ± 3.71	24.72 ± 4.35	24.30%
Naidu	TURJ	0.00039	20.12 ± 4.17	20.34 ± 3.71	24.06 ± 4.08	22.97%
Sahariya	ALGJ	4.20E-08	28.62 ± 4.47	23.91 ± 3.07	28.01 ± 3.72	16.73%
Sahariya	ASHJ	2.50E-11	25.44 ± 3.24	23.91 ± 3.07	23.59 ± 2.88	13.83%
Sahariya	GEOJ	8.80E-05	26.56 ± 5.20	23.91 ± 3.07	26.55 ± 2.22	16.23%
Sahariya	GRKJ	5.70E-09	22.41 ± 3.34	23.91 ± 3.07	23.54 ± 2.82	14.28%
Sahariya	IRNJ	0.0033	28.25 ± 6.45	23.91 ± 3.07	23.05 ± 3.35	12.95%
Sahariya	IRQJ	1.20E-06	23.27 ± 3.64	23.91 ± 3.07	22.95 ± 4.19	12.33%
Sahariya	ITAJ	4.90E-08	23.61 ± 3.70	23.91 ± 3.07	23.76 ± 3.44	11.53%
Sahariya	LIBJ	3.00E-08	25.90 ± 4.01	23.91 ± 3.07	23.10 ± 4.10	12.13%
Sahariya	MORJ	2.40E-08	25.34 ± 3.90	23.91 ± 3.07	24.60 ± 3.17	13.72%
Sahariya	SYRJ	1.60E-14	25.83 ± 3.08	23.91 ± 3.07	24.44 ± 4.52	15.18%
Sahariya	TUNJ	7.10E-05	27.22 ± 5.28	23.91 ± 3.07	24.72 ± 4.35	14.74%
Sahariya	TURJ	0.00013	24.63 ± 4.88	23.91 ± 3.07	24.06 ± 4.08	16.09%
Sahariya	YMNJ	1.10E-06	28.32 ± 4.82	23.91 ± 3.07	28.20 ± 4.32	15.35%
Sahariya	Druze	9.00E-08	27.99 ± 4.45	23.91 ± 3.07	29.67 ± 4.81	12.86%
Sahariya	Bedouin	9.70E-06	25.97 ± 4.71	23.91 ± 3.07	27.48 ± 7.74	9.81%
Sahariya	Palestinian	0.0022	28.50 ± 6.38	23.91 ± 3.07	30.32 ± 6.70	15.45%
Santhal	ASHJ	3.30E-13	27.95 ± 3.49	23.59 ± 3.64	23.59 ± 2.88	15.16%
Santhal	GRKJ	3.10E-14	25.63 ± 3.09	23.59 ± 3.64	23.54 ± 2.82	15.52%

Santhal	IRQJ	1.90E-10	25.06 ± 3.49	23.59 ± 3.64	22.95 ± 4.19	13.36%
Santhal	ITAJ	9.30E-10	26.04 ± 3.74	23.59 ± 3.64	23.76 ± 3.44	13.14%
Santhal	LIBJ	6.30E-11	29.45 ± 4.02	23.59 ± 3.64	23.10 ± 4.10	13.97%
Santhal	MORJ	3.20E-20	28.48 ± 2.88	23.59 ± 3.64	24.60 ± 3.17	15.09%
Santhal	SYRJ	3.70E-14	29.01 ± 3.51	23.59 ± 3.64	24.44 ± 4.52	16.47%
Santhal	TUNJ	2.60E-13	30.09 ± 3.75	23.59 ± 3.64	24.72 ± 4.35	16.30%
Santhal	TURJ	1.40E-12	27.43 ± 3.50	23.59 ± 3.64	24.06 ± 4.08	16.57%
Santhal	YMNJ	1.80E-17	29.09 ± 3.18	23.59 ± 3.64	28.20 ± 4.32	15.57%
Satnami	IRNJ	9.10E-07	20.82 ± 3.52	18.26 ± 2.87	23.05 ± 3.35	13.86%
Satnami	LIBJ	3.70E-13	22.94 ± 2.87	18.26 ± 2.87	23.10 ± 4.10	14.37%
Tharu	GRKJ	3.00E-26	23.70 ± 1.90	20.01 ± 2.32	23.54 ± 2.82	35.05%
Tharu	IRNJ	5.80E-18	24.28 ± 2.62	20.01 ± 2.32	23.05 ± 3.35	20.17%
Tharu	IRQJ	9.60E-11	22.02 ± 1.63	20.01 ± 2.32	22.95 ± 4.19	24.72%
Tharu	ITAJ	2.60E-23	24.93 ± 1.91	20.01 ± 2.32	23.76 ± 3.44	23.13%
Tharu	LIBJ	7.70E-18	25.09 ± 2.44	20.01 ± 2.32	23.10 ± 4.10	20.62%
Tharu	SYRJ	6.60E-22	24.85 ± 1.61	20.01 ± 2.32	24.44 ± 4.52	30.68%
Tharu	TUNJ	3.20E-18	25.28 ± 2.41	20.01 ± 2.32	24.72 ± 4.35	27.90%
Tharu	TURJ	2.60E-32	23.56 ± 1.89	20.01 ± 2.32	24.06 ± 4.08	34.98%
Vaish	GRKJ	7.60E-08	24.48 ± 3.88	22.66 ± 3.19	23.54 ± 2.82	NA
Vaish	IRQJ	0.0022	20.44 ± 4.58	22.66 ± 3.19	22.95 ± 4.19	29.01%
Vaish	ITAJ	0.00055	23.38 ± 4.92	22.66 ± 3.19	23.76 ± 3.44	30.12%
Vysya	GEOJ	6.20E-09	27.65 ± 3.44	22.46 ± 2.99	26.55 ± 2.22	18.09%
Vysya	GRKJ	3.40E-17	24.35 ± 2.69	22.46 ± 2.99	23.54 ± 2.82	16.85%
Vysya	IRNJ	2.60E-11	25.93 ± 2.84	22.46 ± 2.99	23.05 ± 3.35	13.81%
Vysya	IRQJ	1.20E-05	24.21 ± 3.03	22.46 ± 2.99	22.95 ± 4.19	14.29%
Vysya	ITAJ	1.70E-13	23.54 ± 2.91	22.46 ± 2.99	23.76 ± 3.44	13.75%
Vysya	TUNJ	8.20E-10	28.61 ± 3.14	22.46 ± 2.99	24.72 ± 4.35	16.38%
Vysya	TURJ	1.20E-13	27.29 ± 3.36	22.46 ± 2.99	24.06 ± 4.08	19.82%

The P-value given by ALDER is corrected for multiple testing. ALDER estimations for ALDER estimations of admixture proportions using 1-reference population are presented in Table 1 and the estimations here are based on 2-reference populations (see also SI appendix, Materials and Methods). 'NA' is given in the few cases the solution for the admixture proportion was not defined.
