# Supplementary material for: The Genetics of Bene Israel from India Reveals Both Substantial Jewish and Indian Ancestry
Source: PLoS One. 2016 Mar 24;11(3):e0152056. doi: 10.1371/journal.pone.0152056 (PMC4806850; doi:10.1371/journal.pone.0152056)
Supplement: S5 Table — (PDF) [file pone.0152056.s019.pdf]

Table S5. *Q* ratio values between the Bene Israel and other populations.

Population	*Q*-ratio
Kamsali	1.018
Kharia	0.839
Madiga	0.838
Vysya	0.778
Santhal	0.778
Kurumba	0.750
Lodi	0.737
Satnami	0.727
Sahariya	0.721
Bhil	0.714
Vaish	0.710
Hallaki	0.706
Kashmiri_Pandit	0.704
Velama	0.686
Tharu	0.686
IRNJ	0.656
Naidu	0.653
YMNJ	0.630
Mala	0.608
IRQJ	0.592
TUNJ	0.589
SYRJ	0.587
GEOJ	0.581
GRKJ	0.575
DJEJ	0.574
ASHJ	0.565
ALGJ	0.553
TURJ	0.549
LIBJ	0.548
MORJ	0.547
ITAJ	0.528
Meghawal	0.496

Q is defined as $Q = \ln(1 - 2F_{ST}^{AUTO}) / \ln(1 - 2F_{ST}^{X})$ and quantifies the ratio between genetic drift on the autosomes to that on the X chromosome.
