# Supplementary material for: The Genetics of Bene Israel from India Reveals Both Substantial Jewish and Indian Ancestry
Source: PLoS One. 2016 Mar 24;11(3):e0152056. doi: 10.1371/journal.pone.0152056 (PMC4806850; doi:10.1371/journal.pone.0152056)
Supplement: S6 Table — (PDF) [file pone.0152056.s020.pdf]

Table S6. mtDNA haplogroups of Bene Israel samples used in this study

**Haplogroup**	***No. of Samples (%)***
M39	*10 (44.4%)*
M30	*3 (16.7%)*
H	*3 (16.7%)*
M	*2 (11.1%)*

mtDNA haplogroup assignment is based on HaploGrep classification.
